# Transcriptome and Metabolome Analysis Reveals Salt-Tolerance Pathways in the Leaves and Roots of ZM-4 (*Malus zumi*) in the Early Stages of Salt Stress

**DOI:** 10.3390/ijms24043638

**Published:** 2023-02-11

**Authors:** Dajiang Wang, Kun Wang, Simiao Sun, Peng Yan, Xiang Lu, Zhao Liu, Qingshan Li, Lianwen Li, Yuan Gao, Jihong Liu

**Affiliations:** 1 Xinjiang Production and Construction Corps Key Laboratory of Special Fruits and Vegetables Cultivation Physiology and Germplasm Resources Utilization, Agricultural College, Shihezi University, Shihezi 832003, China; 2 National Repository of Apple Germplasm Resources, Research Institute of Pomology, Chinese Academy of Agricultural Sciences (CAAS), Key Laboratory of Horticulture Crops Germplasm Resources Utilization, Ministry of Agriculture and Rural Affairs of the People’s Republic of China, Xingcheng 125100, China; 3 Institute of Horticulture Crops, Xinjiang Academy of Agricultural Sciences, No. 403 Nanchang Road, Urumqi 830091, China; 4 Key Laboratory of Horticultural Plant Biology (MOE), College of Horticulture and Forestry Sciences, Huazhong Agricultural University, Wuhan 430070, China

**Keywords:** integrated analysis, *Malus*, molecular mechanism, salt tolerance

## Abstract

The breeding of salt-tolerant rootstock relies heavily on the availability of salt-tolerant *Malus* germplasm resources. The first step in developing salt-tolerant resources is to learn their molecular and metabolic underpinnings. Hydroponic seedlings of both ZM-4 (salt-tolerant resource) and M9T337 (salt-sensitive rootstock) were treated with a solution of 75 mM salinity. ZM-4’s fresh weight increased, then decreased, and then increased again after being treated with NaCl, whereas M9T337′s fresh weight continued to decrease. The results of transcriptome and metabolome after 0 h (CK) and 24 h of NaCl treatment showed that the leaves of ZM-4 had a higher content of flavonoids (phloretinm, naringenin-7-O-glucoside, kaempferol-3-O-galactoside, epiafzelechin, etc.) and the genes (*CHI*, *CYP*, *FLS*, *LAR*, and *ANR*) related to the flavonoid synthesis pathway showed up-regulation, suggesting a high antioxidant capacity. In addition to the high polyphenol content (L-phenylalanine, 5-O-p-coumaroyl quinic acid) and the high related gene expression (*4CLL9* and *SAT*), the roots of ZM-4 exhibited a high osmotic adjustment ability. Under normal growing conditions, the roots of ZM-4 contained a higher content of some amino acids (L-proline, tran-4-hydroxy-L-prolin, L-glutamine, etc.) and sugars (D−fructose 6−phosphate, D−glucose 6−phosphate, etc.), and the genes (*GLT1*, *BAM7*, *INV1*, etc.) related to these two pathways were highly expressed. Furthermore, some amino acids (S-(methyl) glutathione, N-methyl-trans-4-hydroxy-L-proline, etc.) and sugars (D-sucrose, maltotriose, etc.) increased and genes (*ALD1*, *BCAT1*, *AMY1.1*, etc.) related to the pathways showed up-regulation under salt stress. This research provided theoretical support for the application of breeding salt-tolerant rootstocks by elucidating the molecular and metabolic mechanisms of salt tolerance during the early stages of salt treatment for ZM-4.

## 1. Introduction

“The fruit trees go up the hill and down the beach, do not compete for land with grain and cotton” has become a policy that must be implemented over the long term. The utilization of the “four wastelands” (barren hills, waste valleys, barren hillocks, and desolated beaches) is an important strategic measure for the development of new orchards. This could improve the efficiency of land utilization while simultaneously safeguarding the environment. Soil salinization is one of the primary factors preventing the effective use of land for fruit trees in the “four wastelands” areas. It is predicted that by the year 2050, land will have been degraded by 50% due to soil salinization [1]. The selecting and breeding of salt-tolerant plants is a sustainable way to deal with soil salinization. Up to date, the apple dwarfing rootstocks widely used at home and abroad, M26, MM106, M9, and others, are not tolerant to salt [2]. In order to breed salt-tolerant apple rootstocks, it is necessary to understand the metabolic and molecular mechanisms of apple salt-tolerant resources.

Salt stress impedes plant growth and development, crop productivity, and geographic distribution by imposing ionic toxicity and disrupting the cellular osmotic potential due to the excessive accumulation of Na^+^, affecting apple trees [3,4,5,6,7]. Combating the ion toxicity and osmotic stress caused by salt stress has been demonstrated to occur through the main mechanisms of modulating ionic homeostasis, relieving osmotic stress, and mitigating reactive oxygen species (ROS) accumulation [8,9]. Superoxide dismutase (SOD), peroxidase (POD), ascorbate peroxidase (APX), and catalase (CAT) are the primary enzymes involved in ROS scavenging. Some secondary metabolites can also eliminate ROS when a plant is subjected to salt stress [10,11,12]. Understanding the salt-tolerant plant pathway is a basic requirement for using salt-tolerant plants. The salt overly sensitive (SOS) pathway was the first abiotic stress signaling pathway established in plants. It is essential for ion stress signal transduction and is composed of three key parts: SOS1, SOS2, and SOS3. High Na^+^ stress initiates a calcium signal that activates the SOS3–SOS2 protein kinase complex, which regulates the expression level of *SOS1*, a salt tolerance effector gene encoding a plasma membrane Na^+^/H^+^ antiporter with the function of Na^+^ exclusion [13,14,15,16]. Osmotic signal transduction, which includes the mitogen-activated protein kinase (MAPK) cascade, activated phospholipid signaling, and ABA-dependent osmotic stress signaling, is another important pathway in response to salt stress [11,15,17,18,19]. In addition to the pathways mentioned above, other related genes and substances (such as *AtHKT1*, *IPT5b*, ASP3, ASN1, NIT3, GLN1-1, *CAT1*, *DREB2B*, *CBF3*, *ICE1*, *NHX1*, *MhNPR1*, *MhSHN1*, *MdRGLG3*, *MdNAC047*, cyclic nucleotide-gated channels (CNGCs), MdERF3, MdERF4, MzPIP2;1, MdMYB3, MdY3IP1, MdMYB46, etc.) have been shown to be involved in the process of salt tolerance in plants, including *Malus* plants [20,21,22,23,24,25,26,27,28,29,30,31,32,33,34]. 

There have been no reports of salt-tolerant apple rootstocks being bred with *Malus zumi*, despite the fact that it is the most salt-tolerant *Malus* germplasm resource with a tolerance to 0.6% salt content in soil [27,35,36]. The soil salt content in certain regions in north and northwest China exceeds 0.4% [37,38,39], and these areas are important options for developing new orchards. The calcium-dependent protein kinase (CDPK) pathway was found to be the primary signaling pathway in early studies, but the calcineurin B-like protein-interacting protein kinase (CIPK) and MAPK pathways may also be used during the salt stress response in *Malus zumi* [40]. However, the transcriptional and metabolic mechanism of salt tolerance is still poorly understood, which severely limits its application in the breeding of salt-tolerant rootstocks. As a result of testing *Malus zumi* for salt tolerance, we were able to obtain the superior ZM-4 variety. To learn how ZM-4 reacts to salt stress, we used cutting-edge transcriptome sequencing and metabolomic technology here. The results will provide a theoretical basis for the application of ZM-4 in apple salt-tolerant rootstock breeding.

## 2. Results

### 2.1. Comparison of Salt Tolerance between ZM-4 and M9T337 in Response to Salt Stress

**Figure 1:**
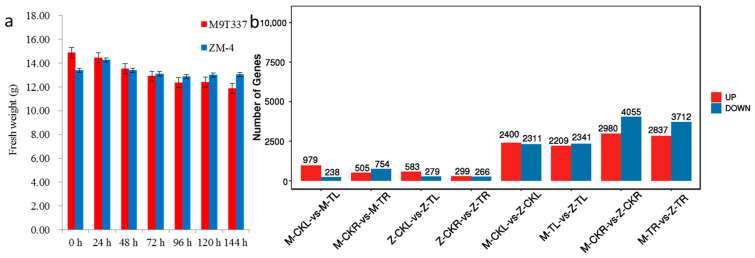
Fresh weight of ZM-4 and M9T337 and differential-expression genes (DEGs) in different combinations under salt stress. (**a**) Fresh weight of ZM-4 and M9T337 after NaCl treatment. (**b**) The number of DEGs in different combinations.

The fresh weights of the hydroponic seedlings of ZM-4 and M9T337 were obtained at 0 h, 24 h, 48 h, 72 h, 96 h, 120 h, and 144 h after the NaCl treatments. The fresh weight of ZM-4 increased from 0 h to 24 h after the NaCl treatments, decreased until 96 h, and then increased again from 96 h to 144 h (13.40 g, 14.26 g, 13.41 g, 13.11 g, 12.87 g, 13.01 g, and 13.05 g at 0 h, 24 h, 48 h, 72 h, 96 h, 120 h, and 144 h, respectively). However, after being exposed to the NaCl solution, M9T337’s fresh weight decreased steadily from 0 h to 144 h (14.88 g, 114.46 g, 13.53 g, 12.90 g, 12.37 g, 12.41 g, and 11.88 g at 0 h, 24 h, 48 h, 72 h, 96 h, 120 h, and 144 h, respectively). The fresh weight of ZM-4 was heavier than that of M9T337 after 48 h of NaCl treatments, despite the fact that the initial fresh weight was lower (Figure 1a). 

### 2.2. Comparison of Transcriptional Profiling between ZM-4 and M9T337 in Response to Salt Stress

In total, 94,658,998, 101,837,367, 86,395,002, 93,715,794, 102,432,303, 122,548,755, 91,373,496, and 115,531,236 clean data points (reads) were identified in the leaves of M9T337 at 0 h (M-CKL), the roots of M9T337 at 0 h (M-CKR), the leaves of M9T337 at 24 h (M-TL), the roots of M9T337 at 24 h (M-TR), the leaves of ZM-4 at 0 h (Z-CKL), the roots of ZM-4 at 0 h (Z-CKR), the leaves of ZM-4 at 24 h (Z-TL), and the roots of ZM-4 at 24 h (Z-TR) from the RNA-seq, respectively. On average, the ratio of the Q30 and GC content in the eight libraries were 94.60% and 46.31%. After mapping to the apple reference genome, the percentages of the total mapped reads were 52.90%, 65.06%, 50.68%, 70.15%, 51.07%, 70.66%, 51.09%, and 70.95% for M-CKL, M-CKR, M-TL, M-TR, Z-CKL, Z-CKR, Z-TL, and Z-TR, respectively (Tables S1 and S2). The Pearson correlation coefficient was used to evaluate sample repeatability, and the results revealed that the repeatability among the biological replicates was reliable (Figure S1).

Genes were identified as novel if they were not already known from comparison to the reference genome. Overall, we were able to pinpoint 41,575 unique genes across all of our samples. The average numbers of genes found in M-CKL, M-CKR, M-TL, M-TR, Z-CKL, Z-CKR, Z-TL, and Z-TR were 33,692, 35,569, 33,633, 35,687, 33,901, 35,716, 33,640, and 35,441, respectively (Table S3). A total of 2307 novel genes were identified from the samples, 126 of which were annotated in the KEGG pathway and 240, 305, and 314 of which were annotated in GO in terms of the cellular components, molecular functions, and biological processes, respectively (Table S4).

#### 2.2.1. DEG Analysis in Leaves in Response to Salt Stress for ZM-4 and M9T337

DEGs were used to identify the key genes involved in salt tolerance. There were 1217, 862, 4711, and 4550 DEGs for M-CKL vs. M-TL, Z-CKL vs. Z-TL, M-CKL vs. Z-CKL, and M-TL vs. Z-TL, respectively (Figure 1b, Table S5). The number of DEGs between ZM-4 and M9T337 with the same treatments was significantly higher than that of the different treatments for the same samples.

**Figure 2:**
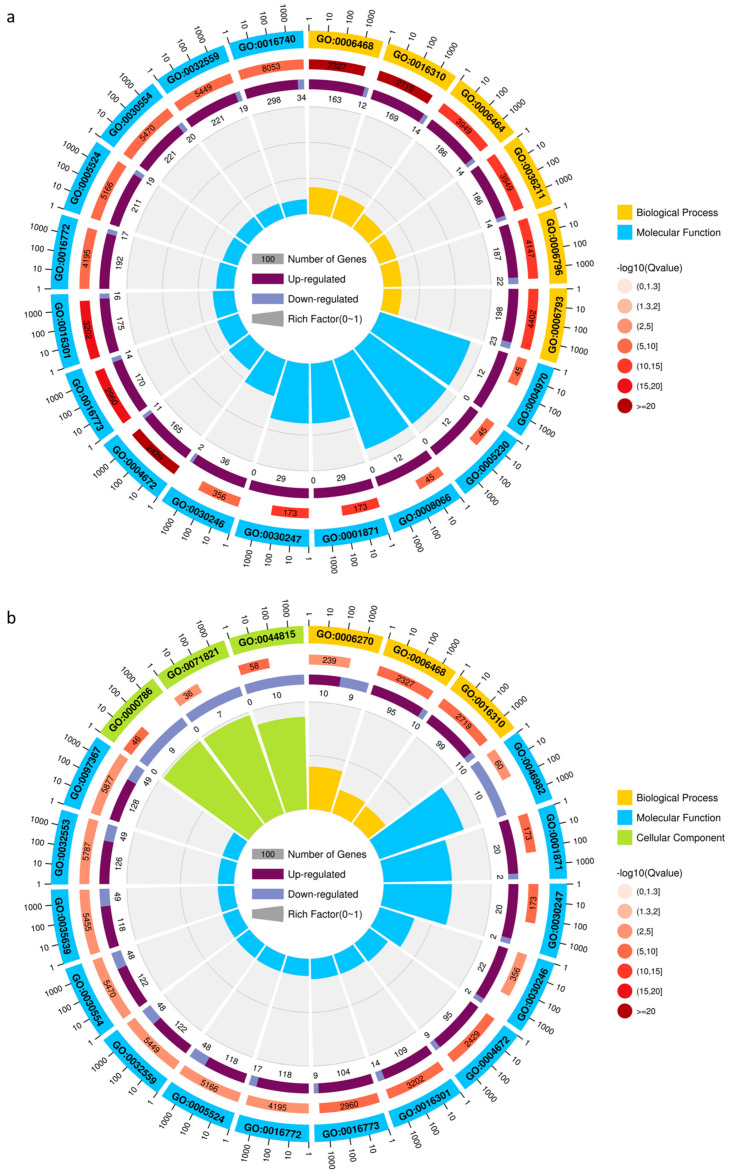

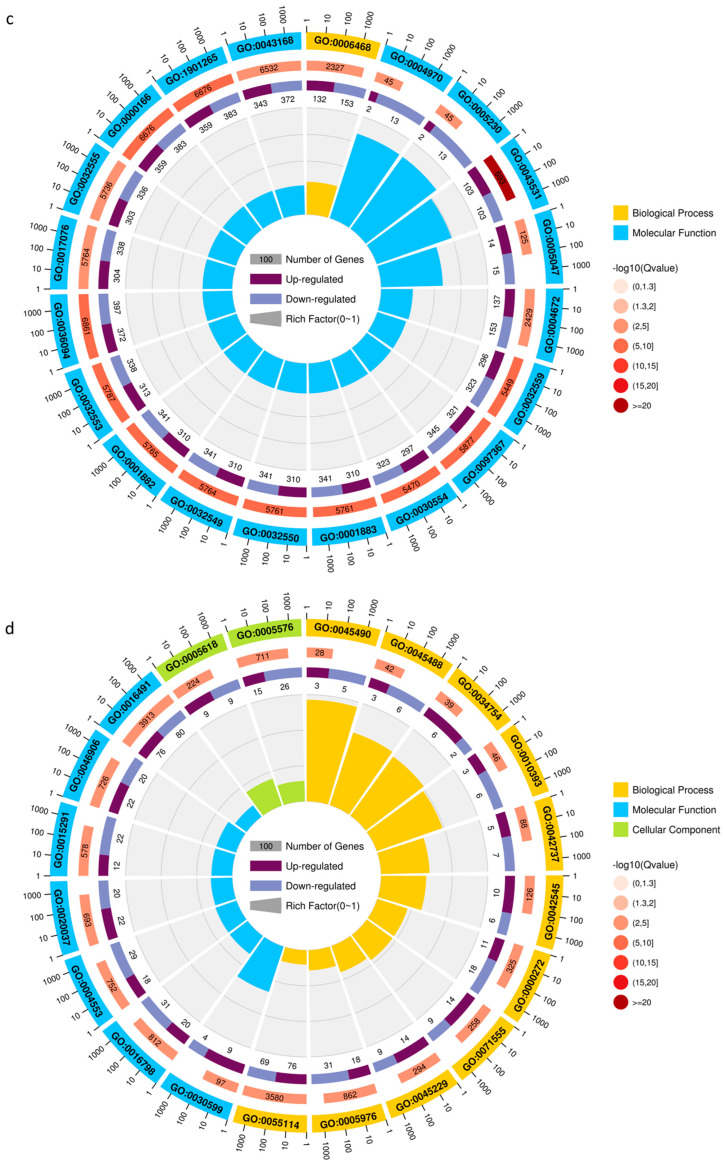

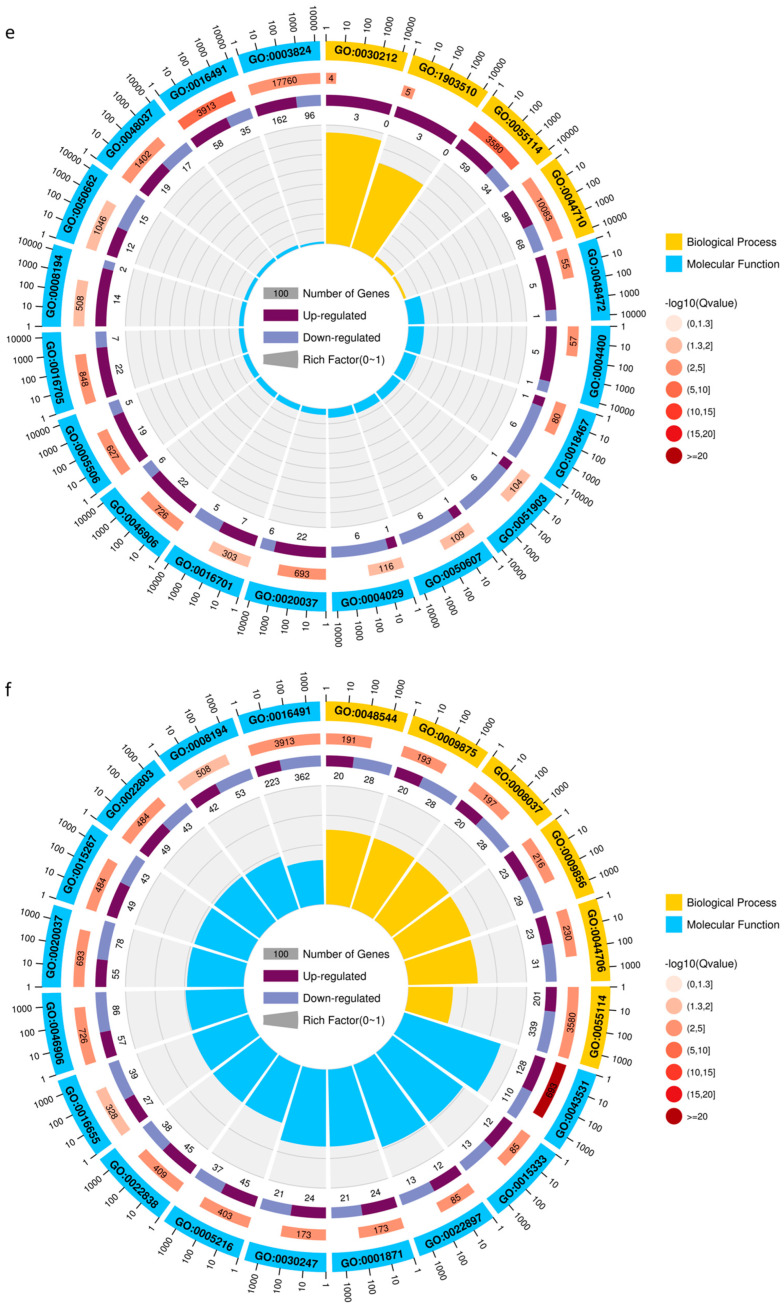
The top 20 GO terms for DEGs in three combinations. (**a**) The top 20 GO terms for DEGs in M-CKL vs. M-TL. (**b**) The top 20 GO terms for DEGs in Z-CKL vs. Z-TL. (**c**) The top 20 GO terms for DEGs in M-TL vs. Z-TL. (**d**) The top 20 GO terms for DEGs in M-CKR vs. M-TR. (**e**) The top 20 GO terms for DEGs in Z-CKR vs. Z-TR. (**f**) The top 20 GO terms for DEGs in M-TR vs. Z-TR.

In comparison with the DEGs at 0 h and 24 h after the NaCl treatment for the GO term in leaves, the top 20 GO terms for the DEGs in M-CKL vs. M-TL were biological processes and molecular functions, with five and fifteen GO terms for them, respectively. The top three terms with a high rich factor were the ionotropic glutamate receptor activity (GO: 0004970), extracellular ligand-gated ion channel activity (GO: 0005230), and glutamate receptor activity (GO: 0008066) (Figure 2a and Figure S2). While the top 20 GO terms for the DEGs in Z-CKL vs. Z-TL differed from those of M9T337, including biological processes, molecular functions, and cellular components, the top three terms with a high rich factor were the nucleosome (GO: 0000786), FANCM-MHF complex (GO: 0071821), and protein heterodimerization activity (GO: 0046982) (Figure 2b and Figure S3). The 20 top GO terms for the DEGs in M-TL vs. Z-TL were biological processes and molecular functions, with the ionotropic glutamate receptor activity (GO: 0004970), extracellular ligand-gated ion channel activity (GO: 0005230), and ADP binding (GO: 0043531) being the top three terms with a high rich factor (Figure 2c and Figure S4).

**Figure 3:**
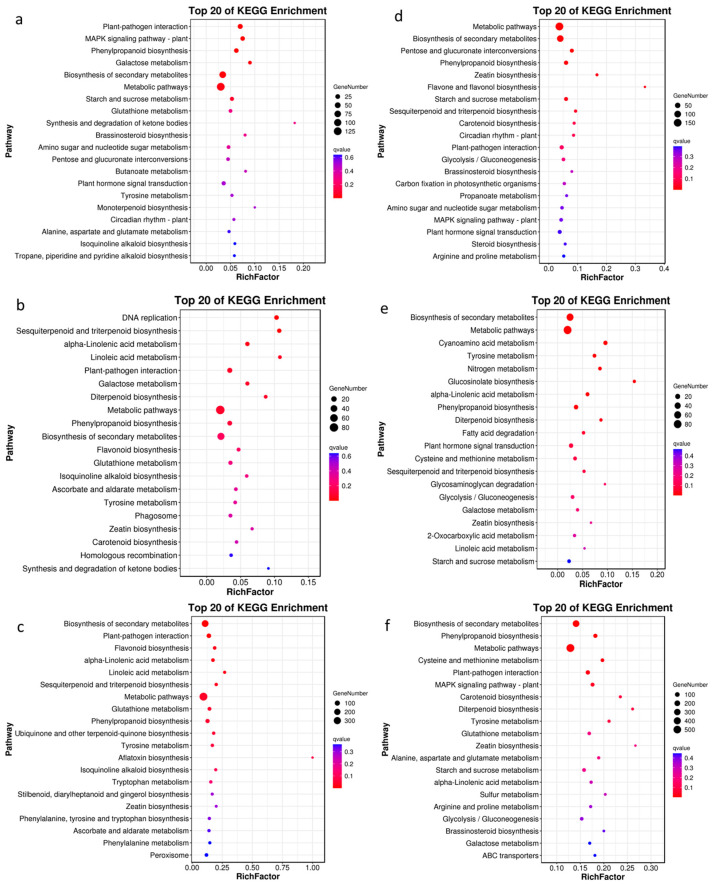
The top 20 KEGG pathways for DEGs in three combinations. (**a**) The top 20 KEGG pathways for DEGs in M-CKL vs. M-TL. (**b**) The top 20 KEGG pathways for DEGs in Z-CKL vs. Z-TL. (**c**) The top 20 KEGG pathways for DEGs in M-TL vs. Z-TL. (**d**) The top 20 KEGG pathways for DEGs in M-CKR vs. M-TR. (**e**) The top 20 KEGG pathways for DEGs in Z-CKR vs. Z-TR. (**f**) The top 20 KEGG pathways for DEGs in M-TR vs. Z-TR.

The top 20 DEG pathways in M-CKL vs. M-TL, according to the KEGG pathway analysis, were metabolism, environmental information processing, and organismal systems. The top three pathways with a high rich factor were the synthesis and degradation of ketone bodies (ko00072), monoterpenoid biosynthesis (ko00902), and galactose metabolism (ko00052) (Figure 3a and Figure S5). The top 20 pathways for the DEGs in Z-CKL vs. Z-TL were different from those of M9T337. These were metabolism, genetic information processing, cellular processes, and organismal systems. The top three pathways with a high rich factor were DNA replication (ko03030), linoleic acid metabolism (ko00591), and sesquiterpenoid and triterpenoid biosynthesis (ko00909) (Figure 3b and Figure S6). The top 20 pathways for the DEGs in M-TL vs. Z-TL were metabolism, cellular processes, and organismal systems. The top two pathways with a high rich factor in M-TL vs. Z-TL were aflatoxin biosynthesis (ko00254) and linoleic acid metabolism (ko00591) (Figure 3c and Figure S7).

**Figure 4:**
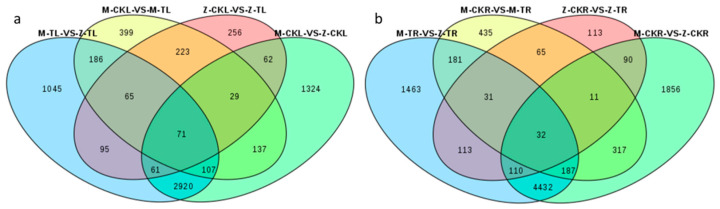
Venn diagrams of DEGs in leaves and roots among different combinations. (**a**) The Venn diagram of DEGs in leaves among different combinations. (**b**) The Venn diagram of DEGs in roots among different combinations.

A Venn diagram among M-TL vs. Z-TL, M-CKL vs. M-TL, Z-CKL vs. Z-TL, and M-CKL vs. Z-CKL was constructed. The results showed that 136 DEGs were coexpressed in the first three combinations, which could be a response to salt stress for both ZM-4 and M9T337. Of these 136 DEGs, two DEGs (MD02G1120200 (*LOX1.5*) and MD03G1138500 (*XA21*)) were enriched in the MAPK signaling pathway, and nine DEGs were enriched in the biosynthesis of secondary metabolites, such as starch and sucrose metabolism, brassinosteroid biosynthesis, phenylpropanoid biosynthesis, and so on (Figure 4a, Table S6). While 95 DEGs were identified in response to salt stress 24 h after the NaCl treatment of ZM-4, the same was not true for M9T337. Among the 95 DEGs, 12 DEGs were enriched in the biosynthesis of secondary metabolites. In addition to the above pathways, flavonoid biosynthesis (MD15G1024100 (*DFR*), MD11G1059500 (*CYP*), and MD10G1311100 (*ANR*)) and ascorbate and aldarate metabolism (MD15G1201000 (*MIOX2*)) are unique pathways that may be the main genes for the salt tolerance of the leaves of ZM-4. 

#### 2.2.2. DEG Analysis of Roots in Response to Salt Stress for ZM-4 and M9T337

A total of 1259, 565, 7035, and 6594 DEGs for M-CKR vs. M-TR, Z-CKR vs. Z-TR, M-CKR vs. Z-CKR, and M-TR vs. Z-TR were identified, respectively (Figure 1b, Table S5). However, the number of DEGs in roots was greater than that in leaves.

The DEGs at 0 h and 24 h after the NaCl treatment were compared for the GO term in roots. The top 20 GO terms for the DEGs in M-CKR vs. M-TR were biological processes, molecular functions, and cellular components, and the top three terms with a high rich factor were the pectin catabolic process (GO:0045490), pectin metabolic process (GO: 00045488), and cellular hormone metabolic process (GO: 0034754) (Figure 2d and Figure S8). While the top 20 GO terms for the DEGs in Z-CKR vs. Z-TR were biological processes and molecular functions, the top three GO terms for the DEGs in Z-CKR vs. Z-TR were the hyaluronan metabolic process (GO: 0030212), mucopolysaccharide metabolic process (GO: 1903510), and threonine-phosphate decarboxylase activity (GO: 0048472) (Figure 2e and Figure S9). The top 20 GO terms for the DEGs in M-TR vs. Z-TR were biological processes and molecular functions, and the top three GO terms for the DEGs in M-TR vs. Z-TR were ADP binding (GO: 0043531), peptide:proton symporter activity (GO: 0015333), and proton-dependent peptide secondary activity (GO: 0022897) (Figure 2f and Figure S10).

The KEGG pathway analysis showed that the top 20 pathways for the DEGs in M-CKR vs. M-TR were metabolism, environmental information processing, and organismal systems. The top two pathways with a high rich factor were flavone and flavonol biosynthesis (ko00944) and zeatin biosynthesis (ko00908) (Figure 3d and Figure S11). The top 20 pathways for the DEGs in Z-CKR vs. Z-TR were different from those of M9T337. These were metabolism and environmental information processing. The top three pathways with a high rich factor were glucosinolate biosynthesis (ko00966), cyanoamino acid metabolism (ko00460), and glycosaminoglycan degradation (ko00531) (Figure 3e and Figure S12). The top 20 GO terms for the DEGs in M-TR vs. Z-TR were metabolism, environmental information processing, and organismal systems, and the top three pathways with a high rich factor in M-TR vs. Z-TR were zeatin biosynthesis (ko00908), diterpenoid biosynthesis (ko00904), and carotenoid biosynthesis (ko00906) (Figure 3f and Figure S13).

The Venn diagram results for roots showed that 63 DEGs were coexpressed in M-TR vs. Z-TR, M-CKR vs. M-TR, and Z-CKR vs. Z-TR, which may be a response to salt stress for both ZM-4 and M9T337. Five DEGs were enriched in the biosynthesis of secondary metabolites, including phenylpropanoid biosynthesis, cysteine and methionine metabolism, and tyrosine metabolism. One DEGs (MD15G1007000 (*P4H3*)) was found to be enriched in arginine and proline metabolism. While 113 DEGs were identified as a response to salt stress 24 h after the NaCl treatment for ZM-4, this was not true for M9T337. Two DEGs (MD01G1158500 (*PYL4*) and MD07G1227100 (*PYL4*)) were enriched in the MAPK signaling pathway, one was enriched in plant hormone signal transduction, and seven DEGs were enriched in the biosynthesis of secondary metabolites, including zeatin biosynthesis, riboflavin metabolism, linoleic acid metabolism, and so on, which may be the main genes for the salt tolerance of the roots of ZM-4 (Figure 4b, Table S6). 

### 2.3. Comparison of Metabolic Profiling between ZM-4 and M9T337 in Response to Salt Stress

**Figure 5:**
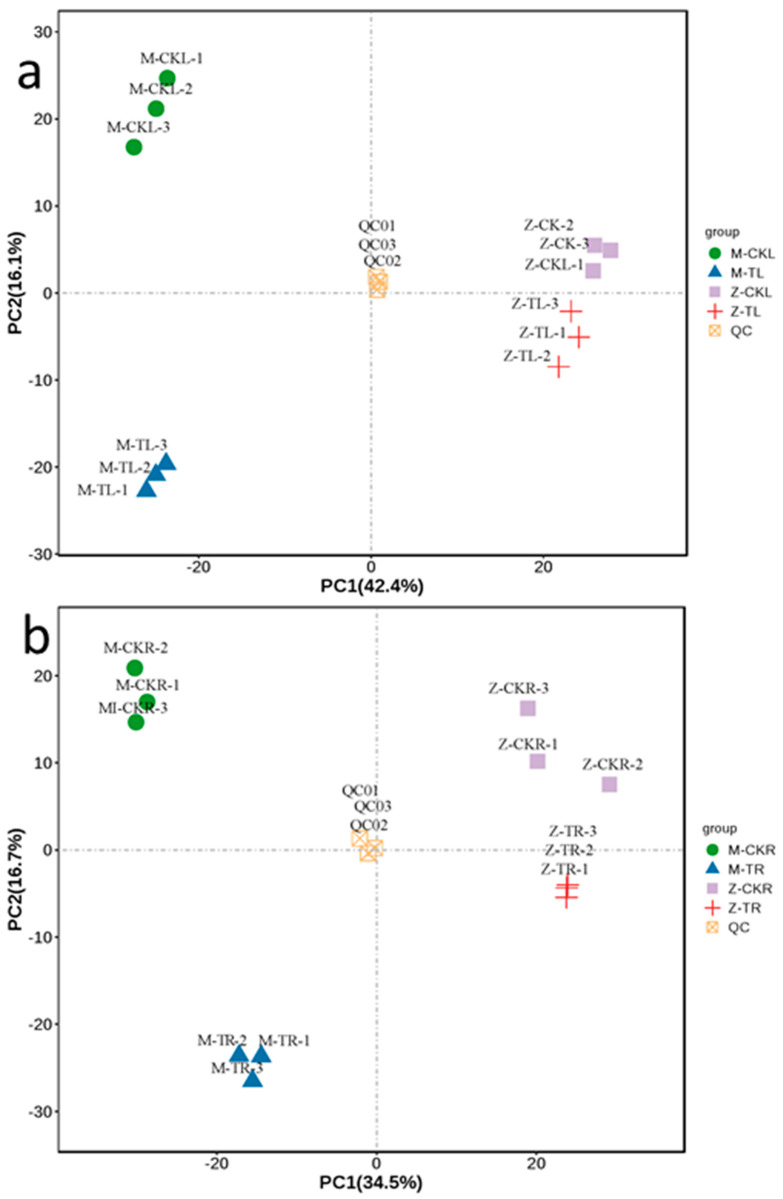
PCA of metabolites in leaves and roots in ZM-4 and M9T337 under salt stress. (**a**) PCA of metabolites in leaves in ZM-4 and M9T337 under salt stress. (**b**) PCA of metabolites in roots in ZM-4 and M9T337 under salt stress.

In total, 1264 metabolites were obtained from the leaves of ZM-4 and M9T337, including alkaloids (85), amino acids and their derivatives (108), flavonoids (331), lignans and coumarins (42), lipids (118), nucleotides and their derivatives (69), organic acids (88), phenolic acids (247), tannins (9), terpenoids (44), and others (123) (Table S7). The roots of ZM-4 and M9T337 yielded a total of 1389 metabolites, including alkaloids (109), amino acids and their derivatives (116), flavonoids (320), lignans and coumarins (40), lipids (149), nucleotides and their derivatives (75), organic acids (104), phenolic acids (284), tannins (13), terpenoids (64), and others (115) (Table S8). The heatmap displayed a clear hierarchical clustering of the samples according to their resources and whether or not they had been treated with NaCl for ZM-4 and M9T337 (Figure S14a,b). The grouping of the QC samples and the separation of the other groups in the PCA plot was indicative of similar metabolic profiles and the overall stability and repeatability of the analysis (Figure 5a,b). 

#### 2.3.1. DAM Analysis in Leaves in Response to Salt Stress for ZM-4 and M9T337

**Figure 6:**
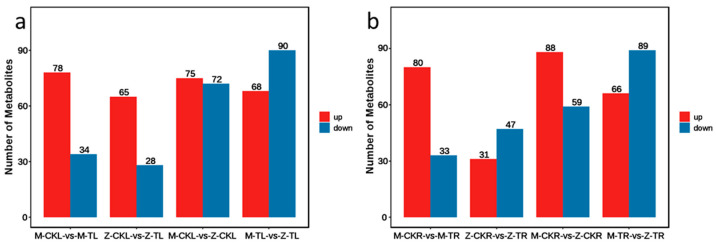
The number of DAMs in leaves and roots of different combinations. (**a**) The number of DAMs in leaves of different combinations. (**b**) The number of DAMs in roots of different combinations.

Differential accumulated metabolites (DAMs) in the leaves showed that there were 112 (78 up and 34 down), 93 (65 up and 28 down), 147 (75 up and 72 down), and 158 (68 up and 90 down) DAMs for M-CKL vs. M-TL, Z-CKL vs. Z-TL, M-CKL vs. Z-CKL, and M-TL vs. Z-TL, respectively (Figure 6a, Table S9).

**Figure 7:**
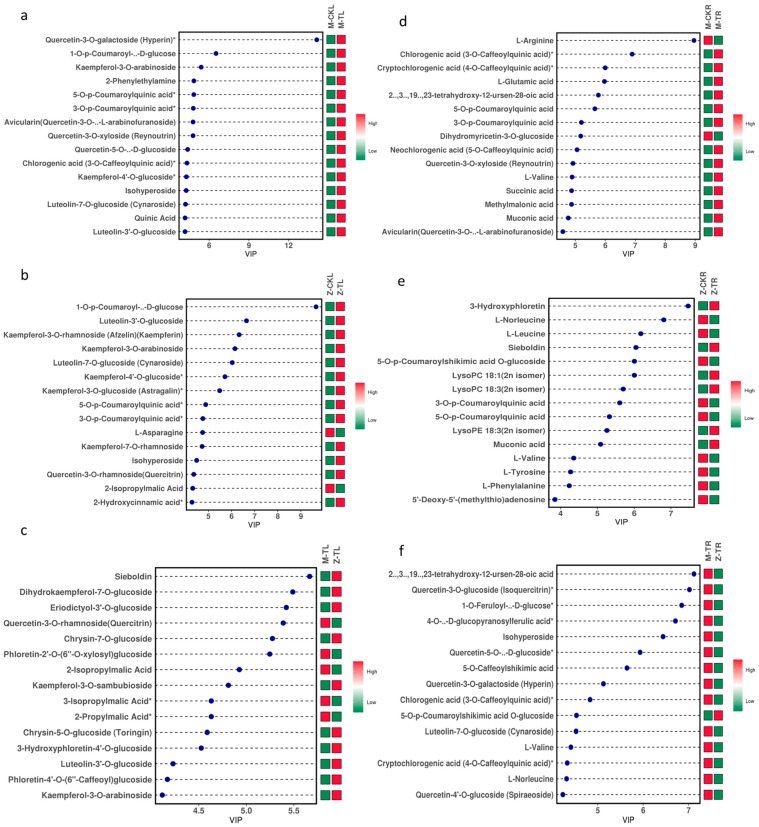
The top 15 DAMs based on the values of VIP in different combinations. (**a**) The top 15 DAMs based on the values of VIP in leaves of M-CKL vs. M-TL. (**b**) The top 15 DAMs based on the values of VIP in leaves of Z-CKL vs. Z-TL. (**c**) The top 15 DAMs based on the values of VIP in leaves of M-TL vs. Z-TL. (**d**) The top 15 DAMs based on the values of VIP in roots of M-CKR vs. M-TR. (**e**) The top 15 DAMs based on the values of VIP in roots of Z-CKR vs. Z-TR. (**f**) The top 15 DAMs based on the values of VIP in roots of M-TR vs. Z-TR.

The KEGG pathway analysis of M-CKL vs. M-TL revealed that DAMs were primarily enriched in the biosynthesis of secondary metabolites, biosynthesis of amino acids, phenylpropanoid biosynthesis, ABC transporters, flavonoid biosynthesis, and so on (Figure S15). The top three VIP values of metabolites were quercetin-3-O-galactoside, 1-O-p-coumaroyl-β-d-glucose, and kaempferol-3-O-arabinoside, and salt stress induced the accumulation of quercetin-3- O -galactoside and 3-hydroxy-3-methylpentane-1,5-dioic acid (Figure 7a, Table S9). The KEGG pathway analysis of Z-CKL vs. Z-TL showed that DAMs were enriched in the biosynthesis of secondary metabolites, biosynthesis of amino acids, flavonoid biosynthesis, 2-oxocarboxylic acid metabolism, and so on (Figure S16). The top three VIP values of metabolites were 1-O-p-coumaroyl-β-d-glucose, luteolin-3’-O-glucoside, and kaempferol-3-O-rhamnoside. However, the accumulation of 3-O-galloyl-d-glucose was induced by salt stress in ZM-4 (Figure 7b, Table S9). The KEGG pathway analysis of M-TL vs. Z-TL showed that the DAMs were similar with Z-CKL vs. Z-TL (Figure S17). The top three VIP values of metabolites were sieboldin, dihydrokaempferol-7-O-glucoside, and eriodictyol-3’-O-glucoside. Daempferol-3-O-robinobioside, kaempferol-3- O -neohesperidoside, and 3’,5,5’,7-tetrahydroxyflavanone-7-O-glucoside were only accumulated in Z-TL, and not in M-TL (Figure 7c, Table S9).

**Figure 8:**
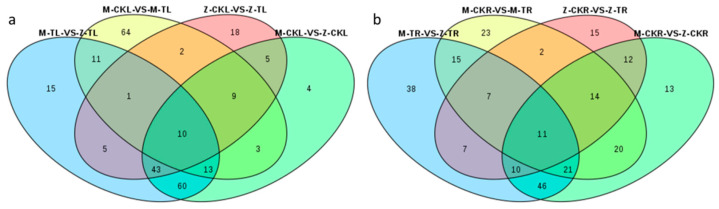
Venn diagrams of DAMs in leaves and roots among different combinations. (**a**) The Venn diagram of DAMs in leaves among different combinations. (**b**) The Venn diagram of DAMs in roots among different combinations.

The Venn diagram results revealed that 11 metabolites were produced in response to salt stress in the leaves of both ZM-4 and M9T337, including four flavonoids (quercetin-3-O-sambubioside, quercetin-3-O-apiosyl(1→2)galactoside, kaempferol-3-O-rhamnosyl(1→2)glucoside, and quercetin-7-O-(6″-malonyl)glucoside), three phenolic acids (5-O-caffeoylshikimic acid, 5-O-p-coumaroylquinic acid, and 3-O-p-coumaroylquinic acid), three organic acids (2-isopropylmalic acid, 3-isopropylmalic acid, and 2-propylmalic acid), and one amino acid and its derivative (L-arginine). Five metabolites were unique in response to the salt stress for ZM-4, including one phenolic acid (2-hydroxycinnamic acid), one flavonoid (quercetin-3-O-robinobioside), one nucleotide and its derivative (2’-deoxyinosine-5’-monophosphate), and two amino acids and their derivatives (L-asparagine and L-aspartic acid) (Figure 8a, Table S10).

#### 2.3.2. DAM Analysis in Roots in Response to Salt Stress for ZM-4 and M9T337

The DAMs in roots showed that there were 113 (80 up and 33 down), 78 (31 up and 47 down), 147 (88 up and 59 down), and 155 (66 up and 89 down) DEMs for M-CKR vs. M-TR, Z-CKR vs. Z-TR, M-CKR vs. Z-CKR, and M-TR vs. Z-TR, respectively (Figure 6b, Table S9).

The KEGG pathway analysis of M-CKR vs. M-TR showed that DAMs were mainly enriched in the metabolic pathways, biosynthesis of secondary metabolites, biosynthesis of antibiotics, biosynthesis of amino acids, and so on (Figure S18). The top three VIP values of metabolites were L-arginine, chlorogenic acid, and cryptochlorogenic acid. LysoPC 18:3 and lysoPC 18:1 were induced to accumulate by salt stress in M9T337 (Figure 7d, Table S9). The KEGG pathway analysis of Z-CKR vs. Z-TR showed that DAMs were enriched in the metabolic pathways, biosynthesis of secondary metabolites, microbial metabolism in diverse environments, biosynthesis of antibiotics, and so on (Figure S19). The top three VIPs of metabolites were 3-hydroxyphloretin, L-norleucine, and L-leucine. L-cysteine and sanguisorbigenin were induced to accumulate by salt stress in ZM-4 (Figure 7e, Table S9). The KEGG pathway analysis of M-TL vs. Z-TL showed that DAMs were enriched in the biosynthesis of secondary metabolites, biosynthesis of amino acids, flavonoid biosynthesis, flavone and flavonol biosynthesis, and so on (Figure S20). The top three VIPs of metabolites were 2α,3α,19α,23-tetrahydroxy-12-ursen-28-oic acid, quercetin-3-O-glucoside, and 1-O-feruloyl-β-d-glucose. Sanguisorbigenin only accumulated in Z-TR (Figure 7f, Table S9). 

The Venn diagram results showed that 18 metabolites were produced in response to salt stress in the roots of both ZM-4 and M9T337, including two flavonoids (isorhamnetin-3-O-glucoside and quercetin-3-O-arabinoside), three phenolic acids (5-O-p-coumaroylquinic acid, 1-O-sinapoyl-β-D-glucose, and propyl 4-hydroxybenzoate), five organic acids (L-malic acid, muconic acid, 3-isopropylmalic acid, 2-propylmalic acid, and 2-isopropylmalic acid), four amino acids and their derivatives (L-glutamic acid, L-leucine, L-proline, and L-valine), one nucleotide and its derivative (adenosine 5’-monophosphate), one alkaloid (pterolactam), one terpenoid (2-hydroxyoleanolic acid) and one other (roseoside). Seven metabolites were specific to the response to salt stress for ZM-4, including one phenolic acid (5-O-p-coumaroylshikimic acid O-glucoside), two flavonoids (isorhamnetin-7-O-glucoside and rhamnetin-3-O-glucoside), one terpenoid (sanguisorbigenin), two organic acids (D-malic acid and 6-aminocaproic acid), and one other (3-dehydro-L-threonic acid) (Figure 8b, Table S10).

### 2.4. Integrated Analysis of the Transcriptome and Metabolome of ZM-4 and M9T337 Responsive to Salt Stress

A coexpression network analysis of the transcriptome and metabolome was performed to further exploit the relationship between DEGs and DAMs in the leaves and roots of ZM-4 and M9T337 under salt stress. The pathway function model and bidirectional orthogonal projections to latent structures model (O2PLS) were carried out to screen the associated genes and metabolites that had an influence on the sample grouping and analysis of the association characteristics. For the leaves, we used 227 DAMs and 6980 DEGs in the association analysis, and for the roots, we used 254 DAMs and 9436 DEGs (Table S11).

#### 2.4.1. Integrated Analysis of the Transcriptome and Metabolome of Leaves Responsive to Salt Stress

The KEGG pathways shared by DAMs and DEGs were examined. A total of 40 pathways were obtained for M-CKL vs. M-TL, including 229 DEGs and 41 DAMs (Tables S12 and S13). The first three pathways were phenylpropanoid biosynthesis, the biosynthesis of secondary metabolites, and metabolic pathways; both the Gene_Pvalue and Gene_Qvalue were less than 0.05, but only the Metabolite_Pvalue of the biosynthesis of secondary metabolites was less than 0.05, making it a candidate pathway for further investigation. A total of 30 pathways were obtained for Z-CKL vs. Z-TL, including 151 DEGs and 41 DAMs. The first three pathways were the same as M-CKL vs. M-TL, while the pathway for which both the Gene_Pvalue and Metabolite_Pvalue were less than 0.05 was flavonoid biosynthesis. A total of 54 pathways were obtained for M-TL vs. Z-TL, including 793 DEGs and 54 DAMs. The first three pathways were the biosynthesis of secondary metabolites, flavonoid biosynthesis, and metabolic pathways. The Gene_Pvalue, Gene_Qvalue, Metabolite_Pvalue, and Metabolite_Qvalue of flavonoid biosynthesis were all less than 0.05, suggesting that this pathway was the major characteristic in the response to salt stress for ZM-4, which included 20 DEGs and 11 DAMs. 

**Figure 9:**
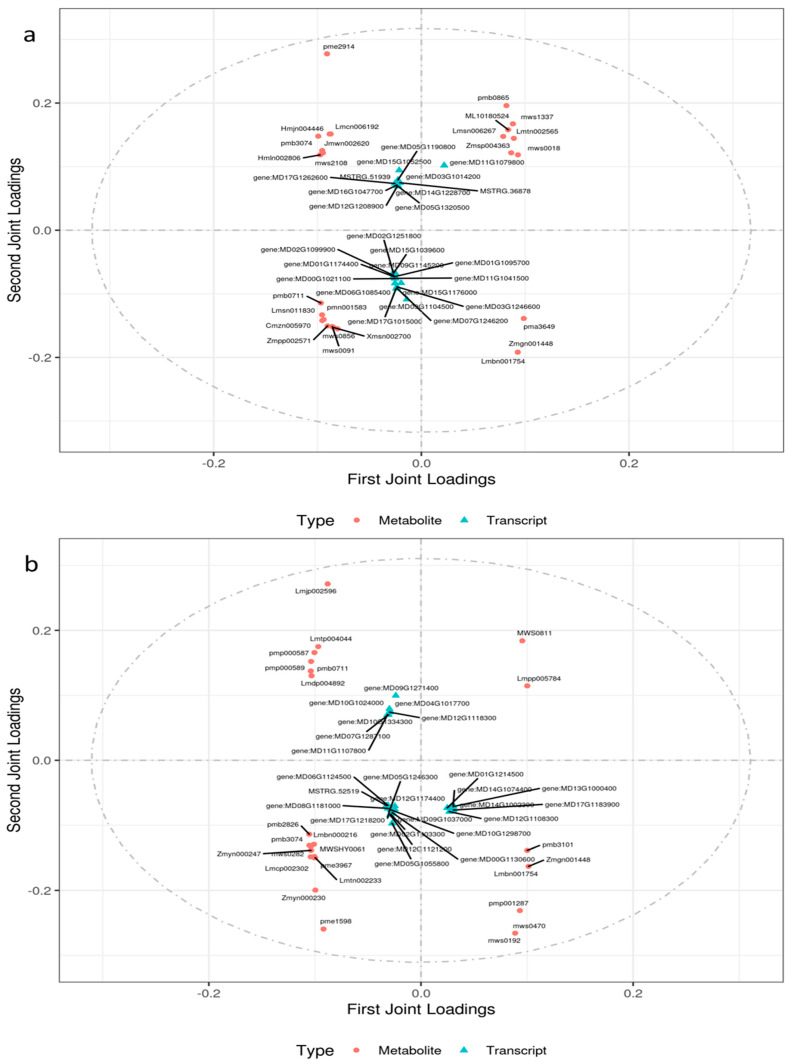

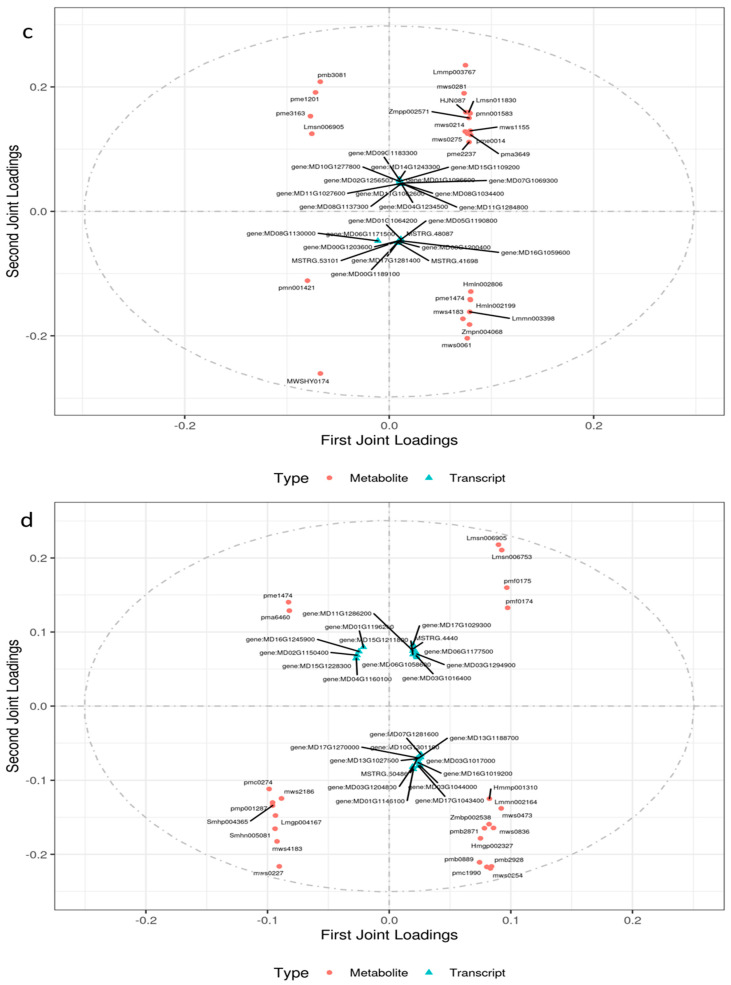

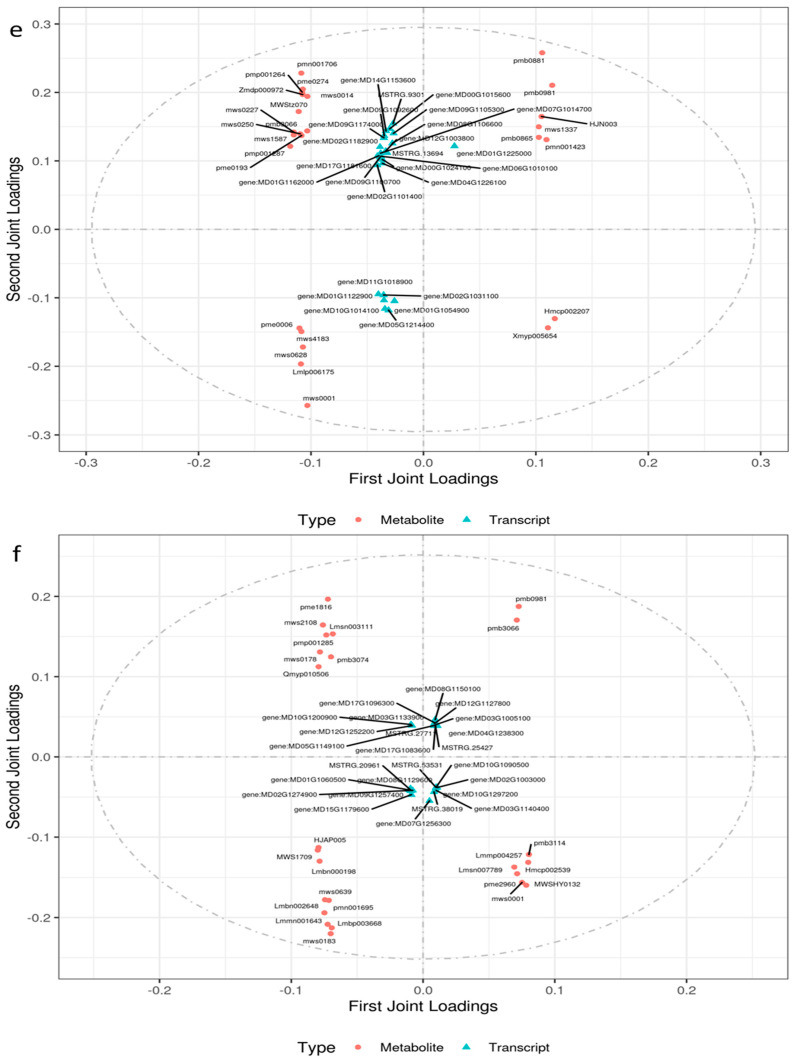
The top 25 joint-loading elements of genes and metabolites in different combinations. (**a**) The top 25 joint-loading elements of genes and metabolites of leaves in M-CKL vs. M-TL. (**b**) The top 25 joint-loading elements of genes and metabolites of leaves in Z-CKL vs. Z-TL. (**c**) The top 25 joint-loading elements of genes and metabolites of leaves in M-TL vs. Z-TL. (**d**) The top 25 joint-loading elements of genes and metabolites of roots in M-CKR vs. M-TR. (**e**) The top 25 joint-loading elements of genes and metabolites of roots in Z-CKR vs. Z-TR. (**f**) The top 25 joint-loading elements of genes and metabolites of roots in M-TR vs. Z-TR.

A total of 1217 DEGs and 112 DAMs, 862 DEGs and 93 DAMs, and 4550 DEGs and 158 DAMs were analyzed by O2PLS for M-CKL vs. M-TL, Z-CKL vs. Z-TL, and M-TL vs. Z-TL, respectively (Table S14). The top 25 DAMs and DEGs were thought to have a high relevance according to the sum of the squares of the first two dimension loading values (Table S15, Figure 9a–c). The first three DAMs and DEGs of M-CKL vs. M-TL were 3-hydroxy-3-methylpentane-1,5-dioic acid, 3-isopropylmalic acid, 2-propylmalic acid, MD09G1104500, MD11G1079800 (RPL4), and MD15G1052500 (NLP3), respectively. The first three DAMs and DEGs of Z-CKL vs. Z-TL were quercetin-3-O-sambubioside, succinic acid, methylmalonic acid, MD09G1271400 (MYB20), MD05G1055800 (GATA5), and MD12G1121200 (MLO3), respectively. The first three DAMs and DEGs of M-TL vs. Z-TL were epicatechin, kaempferol-7-O-glucuronide, glucarate O-phosphoric acid, MD14G1243300 (HDG11), MSTRG.41698 (POL), and MD09G1183300 (GRF5), respectively.

#### 2.4.2. Integrated Analysis of the Transcriptome and Metabolome of Roots Responsive to Salt Stress

The KEGG pathways shared by DAMs and DEGs were examined. A total of 48 pathways were obtained for M-CKR vs. M-TR, including 250 DEGs and 46 DAMs (Tables S12 and S13). The first three pathways were metabolic pathways, the biosynthesis of secondary metabolites, and pentose and glucuronate interconversions, with both the Gene_Pvalue and Gene_Qvalue being less than 0.05, but only the Metabolite_Pvalue and Metabolite_Qvalue of the biosynthesis of secondary metabolites being less than 0.05, so it was the candidate pathway for further investigation. A total of 36 pathways were obtained for Z-CKR vs. Z-TR, including 124 DEGs and 38 DAMs. The first three pathways were the biosynthesis of secondary metabolites, metabolic pathways, and cyanoamino acid metabolism. The Gene_Pvalue, Gene_Qvalue, Metabolite_Pvalue, and Metabolite_Qvalue were less than 0.05. A total of 46 pathways were obtained for M-TR vs. Z-TR, including 1092 DEGs and 42 DAMs. The first three pathways were the biosynthesis of secondary metabolites, phenylpropanoid biosynthesis, and metabolic pathways. The Gene_Pvalue, Gene_Qvalue, Metabolite_Pvalue, and Metabolite_Qvalue of the first two pathways were all less than 0.05, indicating that these pathways are the most important in terms of ZM-4’s response to salt stress. 

A total of 1259 DEGs and 113 DAMs, 565 DEGs and 78 DAMs, and 6549 DEGs and 155 DAMs were analyzed by O2PLS for M-CKR vs. M-TR, Z-CKR vs. Z-TR, and Z-CKR vs. Z-TR, respectively (Table S14). The top 25 DAMs and DEGs were thought to have a high relevance according to the sum of the squares of the first two dimension loading values (Table S15, Figure 9d–f). The first three DAMs and DEGs of M-CKR vs. M-TR were fhloretin-4’-O-(6’’-feruloyl)glucoside, L-leucine, L-histidine, MD01G1146100 (At2g39920), MD17G1029300 (LBD25), and MD17G1043400 (IP5P5), respectively. The first three DAMs and DEGs of Z-CKR vs. Z-TR were LysoPE 18:2, L-asparagine, 2-hydroxyoleanolic acid, MSTRG.9301, MD00G1015600 (fmdA), and MD14G1153600 (rps3), respectively. The first three DAMs and DEGs of M-TR vs. Z-TR were 3,4-dihydroxybenzoic acid, kaempferol-3-O-arabinoside, 2-hydroxycinnamic acid, MD07G1256300 (IRE), MD15G1179600 (RPV1), and MD08G1150100, respectively.

### 2.5. The Salt Tolerance Mechanisms Predicted in Leaves and Roots for ZM-4

**Figure 10:**
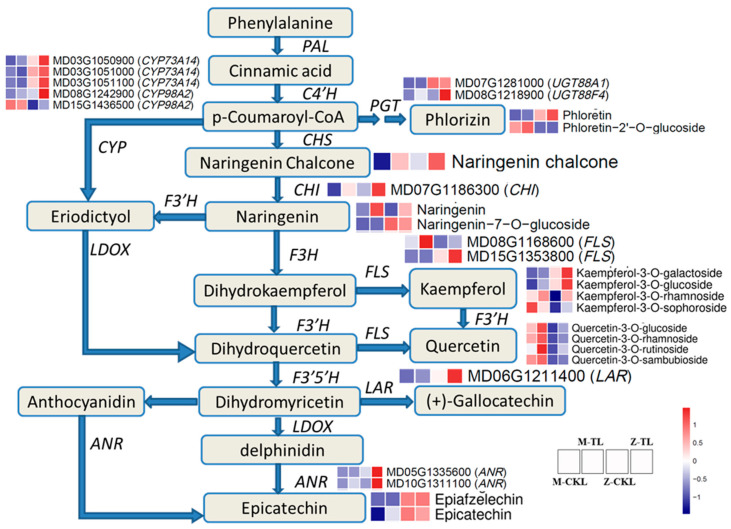
The main salt-tolerance genes and metabolites in phenylpropanoid biosynthesis pathway (PAL, phenylalanine ammonia-lyase; C4′H, cinnamate-4′-hydroxylase; CYP, cytochrome P450; PGT, phydroxybenzoate geranyltransferases; CHS, chalcone synthase; CHI, chalcone isomerase; F3′H, flavonoid-3′-hydroxylase; F3H, flavonoid-3-hydroxylase; LDOX, leucoanthocyanidin dioxygenase; FLS, flavonol synthase; F3′5′H, flavonoid-3′,5′-hydroxylase; LAR, leucoanthocyantin reductase; ANR, anthocyanidin synthase).

The DAMs based on the VIP values (>1) and FC (≥1 or ≤ 0.5) of the metabolites for M-CKL vs. M-TL, Z-CKL vs. Z-TL, and M-TL vs. Z-TL were mainly classified as flavonoids (Table S9). The flavonoid biosynthesis pathway (ko00941) was also enriched by a DEG and DAM joint analysis (Tables S12 and S13). The heatmaps of expression for the DEGs and DAMs of this pathway in M-CKL, M-TL, Z-CKL, and Z-TL were drawn out (Figure 10). Except for quercetin glycosides, kaempferol-3-O-sophoroside, and phloretin-2′-O-glucoside, the other DAMs were upregulated in Z-TL, and the genes related to the DAMs (CHI, CYP, FLS, LAR, and ANR) also showed high expression levels in Z-TL. As a result, the higher expression of the genes and components in the phenylpropanoid biosynthesis pathway was the primary salt-tolerance mechanism in the leaves of ZM-4.

In addition to the flavonoid pathway, the phenylpropanoid biosynthesis pathway was enriched in the roots of M-CKR vs. M-TR, Z-CKR vs. Z-TR, and M-TR vs. Z-TR (Tables S9, S12 and Table S13). 5-O-caffeoyl shikimic acid, chlorogenic acid, and trans-5-O-(p-coumaroyl) shikimate were found in high concentrations in M-TR, whereas 5-O-p-coumaroyl quinic acid and L-phenylalanine were found in high concentrations in T-CKR (Figure S21). The expression of SAT (MD10G1151400), HST (MD17G1224900), and 4CLL9 (MD14G1161200) were induced by salt stress in the roots of ZM-4. The expression of SAT (MD16G1108700 and MD13G1109000) was induced by salt stress in the roots of M9T337. 

**Figure 11:**
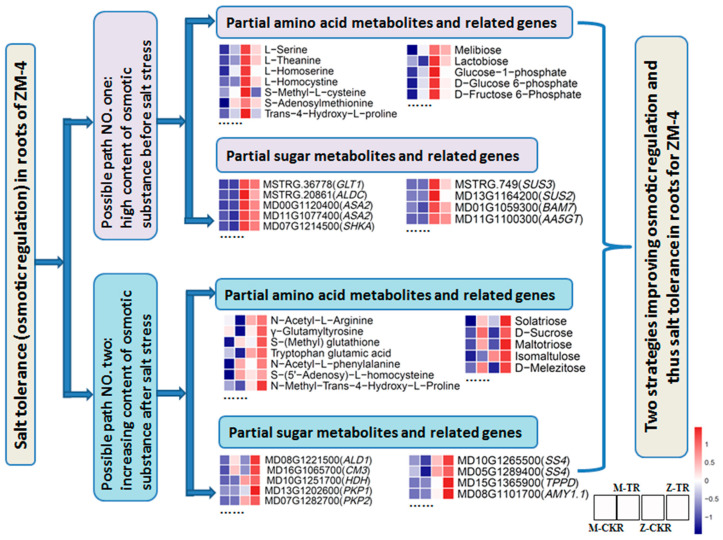
The possible paths of salt stress tolerance in the roots of ZM-4. (Ellipsis indicates the presence of other substances or genes that are not listed).

Furthermore, ZM-4’s fresh weight continued to rise even after 24 h following the NaCl treatment, indicating that osmotic regulation played a crucial role in the response to salt stress. As a result, the osmotic regulating substances, such as amino acids and sugars, were analyzed using a heatmap (Figure 11). There were two paths for maintaining the osmotic pressure. First, it was found that ZM-4, untreated with NaCl, contained a relatively high concentration of various compounds, such as L-proline, tran-4-hydroxy-L-prolin, L-glutamine, L-asparagine, cis-aconitic acid, glutaric acid, argininosuccinic acid, D−fructose 6−phosphate, D−glucose 6−phosphate, D−sorbitol, and so on (Figure 11, Tables S5 and S8). The genes related to these metabolites were highly expressed, such as MSTRG.36778 (GLT1), MSTRG.20861 (ALDC), MD11G1100300 (AA5GT), MD01G1059300 (BAM7), MD14G1028500 (INV1), MD13G1164200 (SUS2), and MSTRG.749 (SUS3). On the other hand, some substances were increased after the NaCl treatment. For example, S-(methyl) glutathione, N-methyl-trans-4-hydroxy-L-proline, 2-hydroxyethylphosphonic acid, 3-guanidinopropionic acid, allantoin, D-sucrose, maltotriose, melezitose, maltitol, sucrose−6−phosphate, isomaltulose, and others were examples. The genes involved in the synthesis of these metabolites included MD08G1221500 (ALD1), MD08G1075600 (BCAT1), MD16G1065700 (CM3), MD04G1091900 (METK4), MD13G1202600 (PKP1), MD08G1101700 (AMY1.1), MD06G1066600 (INVA), MD10G1265500 (SS4), MD05G1289400 (SS4), and MD15G1365900 (TPPD) (Figure 11, Tables S5 and S8).

## 3. Discussion

High Na^+^ concentrations in the soil generate hyperosmotic conditions that can severely impact plant nutrients and water uptake, as well as cause leaf withering and, eventually, plant death [41,42]. When exposed to saline soil, the first stress experienced by a plant is osmotic stress, which impedes plant growth and development [11,43,44,45]. According to the findings of this study, the fresh weight of M9T337 fell following the NaCl treatment, whereas the fresh weight of ZM-4 increased from 0 h to 24 h, decreased until 96 h, and then climbed again from 120 h to 144 h. Although lower at 0 h, the fresh weight of ZM-4 was higher than that of M9T337 after 72 h. This phenotype indicates that ZM-4 was less influenced by the osmotic stress from 0 h to 24 h, even in the later stages. This phenotype demonstrates that ZM-4 was less influenced by the osmotic stress after 24 h. ZM-4 was shown to be more salt tolerant because it both naturally contained and was induced to produce more osmotic-regulating substances in response to salt stress. 

The mapped reads ranged from 51.07% to 70.95% among the eight libraries, yielding a total of 41,575 genes and resulting in the identification of 2307 novel genes. The total number of genes was less than that of the GDDH13 (reference genome) [46] and the HFTH1 [47]. The ratio of mapped reads was slightly lower and more novel genes were discovered, which could be attributed to ZM-4’s and M9T337’s genetic link being distantly related to golden delicious (GDDH13), the level of which was lower in the leaves than in the roots. This was primarily because leaves have fewer genes than roots. It was preferable to use the *Malus zumi* genome de novo assembly as the reference genome.

ROS are produced as a result of high salinity stress and are largely detoxified by the enzymes SOD, CAT, APX, and POD [44,48,49]. This study discovered that the genes of *APX3* (MD15G1242900, MD15G1243000, MD00G1173600, and MD05G1120800), *APXT* (MD00G1029300 and MD00G1029400), *SOD* (MD01G1164700), *SODA* (MD07G1232200), and *CAT1* (MD06G1008600) were found to be highly expressed in the leaves of ZM-4. It could be one of the reasons for the salt tolerance of ZM-4. Along with these enzymes, flavonoids also perform a major function in ROS scavenging [50,51,52,53]. DEGs and DAMs were found to be more enriched in the flavonoid pathway in ZM-4 leaves. *CHI*, *CYP*, *FLS*, *LAR*, and *ANR* were up-regulated in the leaves of ZM-4, and high accumulations of phloretin, naringenin chalcoe, kaempferol-3-O-galactoside, kaempferol-3-O-glucoside, epiafzelechin, and epicatechin were found. As a result, the flavonoid pathway may play a key role in the response of ZM-4 leaves to salt stress. 

The principal locations of salt stress response are the roots, and the initial salt reaction is derived primarily from the roots. Roots are severely damaged by stress-induced ROS excess [54,55]. The POD, SOD, and CAT activities were higher in *Malus zumi* roots than in the leaves [46]. Polyphenols were also crucial in reducing ROS, alongside antioxidant enzymes and flavonoids [56,57,58]. In the roots of ZM-4, the content of L-phenylalanine and 5-O-p-coumaroyl quinic acid accumulated, and MD17G1224900 (*HST*), MD10G1161400 (*SAT*), and MD14G1161200 (*4CLL9*) were highly expressed in the roots of ZM-4. This suggests that ZM-4’s salt tolerance is in part due to its roots’ antioxidant capacity.

Not only can reactive oxygen species (ROS) damage root function, but so does osmotic dysregulation. Roots are not able to absorb water if subjected to osmotic stress [59]. In this study, the fresh weight of ZM-4 increased before 24 h following the NaCl treatment, while that of M9T337 kept falling afterward. This suggests that M9T337 was subjected to higher degrees of osmotic stress than ZM-4, implying that ZM-4’s roots contained more osmotic-regulating substances. Soluble sugars, palmitoleic acid, D-arginine, pheophytin a, rutin, and vanillin were found to be crucial in promoting salt-stressed plant growth [60,61,62]. Under normal conditions, ZM-4 contained a high concentration of L-proline, tran-4-hydroxy-L-prolin, L-glutamine, lactobiose, D-glucosamine, and other compounds. It can maintain a high osmotic potential in the roots and absorb nutrients and water properly in the early phases of salt stress; this may be the one of the main reasons for its tolerance to salt stress. Furthermore, some other amino acids and sugars were induced to accumulate after salt stress, such as S-(methyl) glutathione, N-methyl-trans-4-hydroxy-L-proline, D-sucrose, planteose, maltotriose, and so on, and the genes (*PKP1*, *PFK2*, *GLT1*, *SUS2*, *SUS3*, *SS4*, etc.) related to these substances were up-regulated. This increased the roots’ ability to regulate the osmotic pressure. This demonstrates that plant hormone signal transduction was enriched in the leaves, while starch and sucrose metabolism, as well as proline biosynthesis, were enriched in trifoliate orange roots [10]. Sucrose’s reactions as a signaling molecule were crucial to *Malus halliana* in maintaining an osmotic equilibrium and eliminating ROS during salt stress. It directly mediated the accumulation of D-phenylalanine, tryptophan, and alkaloids (vindoline and ecgonine), as well as the expression of aspartate and glutamate-related proteins (*ASP3*, *ASN1*, *NIT4*, and *GLN1−1*) [24]. *Malus robusta*’s tolerance to salt, alkali, and salt–alkali stress was conferred by SNP182G on *MdRGLG3*, which converted a leucine to an arginine at the vWFA domain [32]. This could be the principal salt-tolerance mechanism in roots for ZM-4, because it had a high content of some amino acids and sugars and a high expression of related genes on its own. Additionally, salt stress induced the accumulation and expression of other amino acids, sugars, and related genes.

## 4. Materials and Methods

### 4.1. Plant Materials

The experiment was conducted in an artificial climate chamber at 26–28 °C with a photoperiod of 16 h light/8 h dark. Tissue culture seedlings of ZM-4 and M9T337 were transplanted to point trays after rooting for a month in the tissue culture bottles, and then the seedlings were transplanted to hydroponic tanks with Hoagland nutrient solution (Ca(NO_3_)_2_·4H_2_O: 0.005 mol/L, KNO_3_: 0.005 mol/L, MgSO_4_·7H_2_O: 0.002 mol/L, KH_2_PO_4_: 0.001 mol/L, H_3_BO_3_: 2.86 mg/L, MnCl_2_·4H_2_O: 1.81: mg/L, ZnSO_4_·7H_2_O: 0.22 mg/L, CuSO_4_·5H_2_O: 0.08 mg/L, H_2_MoO_4_·H_2_O/Na_2_MoO_4_·2H_2_O: 0.02/0.03 mg/L, and FeEDTA solution: 2 mL) when they were approximately 30 cm tall in the point trays. The hydroponic seedlings were transplanted into new Hoagland nutrient solution with 75 mM NaCl one week later. The samples of leaves and roots were collected 0 h and 24 h after the NaCl treatment. They were frozen in liquid nitrogen for a short time, and then were stored in a −80 °C ultra-low-temperature refrigerator until the transcriptomic and metabolomic analysis. 

### 4.2. Phenotype of the Samples

The fresh weight was measured at 0 h, 24 h, 48 h, 72 h, 96 h, 120 h, and 144 h after the 75 mM NaCl treatment. Three seedlings were used to collect the fresh weight for the hydroponic seedlings of ZM-4 and M9T337. The average fresh weight of three seedlings was used as the phenotype to evaluate the salt tolerance of ZM-4 and M9T337. 

### 4.3. Transcriptome Analysis

#### 4.3.1. RNA Quantification and Qualification

The total RNA was extracted using a Trizol reagent kit (Invitrogen, Carlsbad, CA, USA) according to the manufacturer’s protocol. The RNA quality was assessed on an Agilent 2100 Bioanalyzer (Agilent Technologies, Palo Alto, CA, USA) and checked using RNase free agarose gel electrophoresis. After the total RNA was extracted, the rRNAs were removed to retain mRNAs. The enriched mRNAs were fragmented into short fragments by using a fragmentation buffer and reverse-transcribed into cDNA with random primers. Second-strand cDNA was synthesized with DNA polymerase I, RNase H, dNTP (dUTP instead of dTTP), and buffer. Next, the cDNA fragments were purified with a QiaQuick PCR extraction kit (Qiagen, Venlo, The Netherlands), the ends were repaired, poly (A) was added, and the fragments were ligated to Illumina sequencing adapters. Then, UNG (uracil-N-glycosylase) was used to digest the second-strand cDNA. The digested products were size-selected by agarose gel electrophoresis, amplified with PCR, and sequenced using Illumina HiSeqTM 4000 by Gene Denovo Biotechnology Co. (Guangzhou, China).

#### 4.3.2. Filtering of Clean Reads

The reads obtained from the sequencing machines included raw reads containing adapters or low-quality bases, which would affect the following assembly and analysis. Thus, to obtain high-quality clean reads, the reads were further filtered by fastp (version 0.18.0) [63]. The parameters were as follows: (1) reads containing adapters were removed; (2) reads containing more than 10% of unknown nucleotides (N) were removed; and (3) low-quality reads containing more than 50% low-quality (Q-value ≤ 20) bases were removed. 

The short-reads alignment tool Bowtie2 (version 2.2.8) [64] was used for mapping the reads to the ribosome RNA (rRNA) database. The rRNA-mapped reads were then removed. The remaining reads were further used in the assembly and analysis of the transcriptome.

#### 4.3.3. Novel Transcript Identification and Annotation

An index of the reference genome was built, and paired-end clean reads were mapped to the reference genome (GDDH13 v 1.1) [46] using HISAT2 (version 2.1.0) [65] with “-rna-strandness RF” and other parameters set as a default. 

The reconstruction of transcripts was carried out with the software Stringtie (version 1.3.4) [66,67], which, together with HISAT2, allows biologists to identify new genes and new splice variants of known ones. To identify the new transcripts, all of the reconstructed transcripts were aligned to the reference genome and divided into twelve categories by using Cuffcompare. Transcripts with one of the classcodes “u, i, j, x, c, e, o” were defined as novel transcripts. We used the following parameters to identify reliable novel genes: a transcript length of longer than 200 bp and an exon number of more than 1. Novel transcripts were then aligned to the Nr, KEGG, and GO databases to obtain the protein functional annotation. 

#### 4.3.4. Quantification of Transcript Abundance

The transcript abundances were quantified by the software StringTie in a reference-based approach. For each transcription region, an FPKM (fragment per kilobase of transcript per million mapped reads) value was calculated to quantify its expression abundance and variations using the StringTie software. 

The FPKM formula is shown as follows:

$$\mathrm{FPKM}=\frac{10^{6}C}{\mathrm{NL}/10^{3}}$$

where FPKM(A) is the expression of transcript A, C is the number of fragments mapped to transcript A, N is the total number of fragments that were mapped to reference genes, and L is the number of bases on transcript A. The FPKM method was able to eliminate the influence of different transcript lengths and the amount of sequencing data on the calculation of transcript expression. Therefore, the calculated transcript expression can be directly used for comparing the difference in transcript expression among the samples. 

#### 4.3.5. Differentially Expressed Transcript (DEG) Analysis

The differentially expressed transcripts of coding RNAs were analyzed. An RNA differential expression analysis was performed by the DESeq2 [68] software between two different groups (and by edgeR [69] between two samples). The genes/transcripts with a false discovery rate (FDR) parameter below 0.05 and an absolute fold change ≥ 2 were considered differentially expressed genes/transcripts. Differentially expressed coding RNAs were then subjected to an enrichment analysis of GO functions and KEGG pathways.

### 4.4. Metabolic Analysis

#### 4.4.1. Chemicals and Reagents

All chemicals and reagents were of analytical grade. Methyl alcohol, acetonitrile, and ethyl alcohol were purchased from Merck Company, Germany. Milli-Q system (Millipore Corp., Bedford, MA, USA) ultrapure water was used throughout the study. Authentic standards were purchased from BioBioPha Co., Ltd.(Kunming, China) and Sigma-Aldrich (St. Louis, MO, USA). 

#### 4.4.2. Sample Preparation and Extraction

The freeze-dried samples were crushed using a mixer mill (MM 400, Retsch) with a zirconia bead for 1.5 min at 30 Hz. Then, 100 mg of powder was weighed and extracted overnight at 4 °C with 1.0 mL of 70% aqueous methanol containing 0.1 mg/L lidocaine for an internal standard. Following centrifugation at 10,000× *g* for 10 min, the supernatant was absorbed and filtrated (SCAA-104, 0.22 μm pore size; ANPEL, Shanghai, China, www.anpel.com.cn/ (accessed on 9 May 2022)) before the LC–MS/MS analysis. Quality control (QC) samples were mixed by all samples to assess the reproducibility of the whole experiment.

#### 4.4.3. AB Sciex QTRAP4500 (UPLC) Analysis

The compounds extracted were analyzed using an LC-ESI-MS/MS system (UPLC, Shim-pack UFLC SHIMADZU CBM30A, http://www.shimadzu.com.cn/ (accessed on 19 April 2022); MS/MS (Applied Biosystems 6500 QTRAP) [70].

An amount of 2 μL of the samples were injected onto a Waters ACQUITY UPLC HSS T3 C18 column (2.1 mm × 100 mm, 1.8 μm) operating at 40 °C and with a flow rate of 0.4 mL/min. The mobile phases used were acidified water (0.04% acetic acid) (Phase A) and acidified acetonitrile (0.04% acetic acid) (Phase B). The compounds were separated using the following gradient: 95:5 Phase A/Phase B at 0 min; 5:95 Phase A/Phase B at 11.0 min; 5:95 Phase A/Phase B at 12.0 min; 95:5 Phase A/Phase B at 12.1 min; and 95:5 Phase A/Phase B at 15.0 min. The effluent was connected to an ESI-triple quadrupole-linear ion trap (Q TRAP)–MS.

The LIT and triple quadrupole (QQQ) scans were acquired on a triple quadrupole-linear ion trap mass spectrometer (Q TRAP), AB Sciex QTRAP6500 system, equipped with an ESI-Turbo Ion-Spray interface, operating in a positive ion mode and controlled by the Analyst 1.6.1 software (AB Sciex). The operation parameters were as follows: ESI source temperature, 500 °C; ion spray voltage (IS), 5500 V; curtain gas (CUR), 25psi; and the collision-activated dissociation (CAD) was set to the highest. The QQQ scans were acquired as MRM experiments with an optimized declustering potential (DP) and collision energy (CE) for each individual MRM transition. The *m*/*z* range was set between 50 and 1000.

#### 4.4.4. Data Pre-Processing and Metabolite Identification

The data filtering, peak detection, alignment, and calculations were performed using the Analyst 1.6.1 software. To produce a matrix containing fewer biased and redundant data, peaks were checked manually for a signal/noise (s/n) > 10 and in-house software written in Perl was used to remove the redundant signals caused by different isotopes; in-source fragmentation; K^+^, Na^+^, and NH4^+^ adducts; and dimerization. To facilitate the identification/annotation of metabolites, an accurate *m*/*z* for each Q1 was obtained. The total ion chromatograms (TICs) and an extracted ion chromatogram (EICs or XICs) for the QC samples were exported to give an overview of the metabolite profiles of all samples. The area of each chromatographic peak was calculated. The peaks were aligned across the different samples based on the spectral pattern and retention time. The metabolites were identified by searching an internal database and public databases (MassBank, KNApSAcK, HMDB [71], MoTo DB, and METLIN [72]) and comparing the *m*/*z* values, the RT, and the fragmentation patterns with the standards.

#### 4.4.5. Multivariate Statistical Analysis

For a preliminary visualization of the differences between different groups of samples, an unsupervised dimensionality reduction method principal component analysis (PCA) was applied to all samples using R package models. 

The OPLS-DA model was further validated by cross-validation and 200 permutation tests [73]. For cross-validation, the data were partitioned into seven subsets, where each of the subsets was then used as a validation set. R^2^ indicated the total variation in the data matrix that was explained by the model. The predictive ability (Q^2^) values represented the most recognized diagnostic statistical parameter to validate the OPLS-DA model in metabolomics. An acceptable predictive model was considered for a Q^2^ value greater than 0.4. A good predictive model was considered for a Q^2^ value greater than 0.9. A permutation test randomly permuted class labels 200 times and then produced a distribution of R^2′^ values and Q^2′^ values. In essence, a reliable model should yield significantly larger R^2^ and Q^2^ values compared to the R^2′^ and Q^2′^ values generated from random models using the same dataset.

The loadings from (O) PLS were the directions of projection with respect to the original variables. Variables whose loadings were far away from the origin in a loadings plot might be inferred to have the greatest contribution to class separation.

#### 4.4.6. Differential Metabolite Analysis

A variable importance in projection (VIP) score of the (O) PLS model was applied to rank the metabolites that best distinguished between the two groups. The threshold of the VIP was set to 1. In addition, a *t*-test was also used as a univariate analysis for screening differential metabolites. Those with a *p*-value of the *t*-test < 0.05 and a VIP ≥ 1 were considered differential metabolites between two groups.

#### 4.4.7. KEGG Pathway Analysis

KEGG is the major public pathway-related database that includes not only genes, but also metabolites [74]. The metabolites were mapped to KEGG metabolic pathways for a pathway analysis and an enrichment analysis. A pathway enrichment analysis identified significantly enriched metabolic pathways or signal transduction pathways in differential metabolites by comparing with the whole background. The calculating formula is as follows:

$$P=1-\overset{m-1}{\underset{i=0}{\sum }}\frac{\left(\begin{array}{c} M\\ i \end{array}\right)\left(\begin{array}{c} N-M\\ n-i \end{array}\right)}{\left(\begin{array}{c} N\\ n \end{array}\right)}$$

Here, *N* is the number of all metabolites with KEGG annotation, *n* is the number of differential metabolites in *N*, *M* is the number of all metabolites annotated to specific pathways, and m is number of differential metabolites in *M*. The calculated *p*-value was obtained through an FDR correction, taking FDR ≤ 0.05 as a threshold. Pathways meeting this condition were defined as significantly enriched pathways in differential metabolites.

### 4.5. Joint Analysis of Transcriptome and Metabolome

#### 4.5.1. Pathway Model

KEGG pathway maps are the linking of genomic or transcriptomic contents of genes to chemical structures of endogenous molecules, thus providing a method of performing an integration analysis of genes and metabolites. All differentially expressed genes and metabolites in this study were mapped to the KEGG pathway database to obtain their links in metabolic pathways.

#### 4.5.2. O2PLS Model

In order to integrate the transcriptomic and metabolomic data, we performed a two-way orthogonal PLS (O2PLS) analysis [75]. This method decomposes the variation present in the two data matrices into three parts: the joint variation between the two datasets, the orthogonal variation that is unique to each dataset, and noise. The model assumes that some latent variables are responsible for the variation in the joint and orthogonal parts. O2PLS models were calculated using the OmicsPLS package of R. To determine the optimal number of components, the proposed alternative cross-validation procedure was utilized [76]. The best models were used for the integration analysis.

## 5. Conclusions

For this experiment, we tested the hydroponic seedlings of ZM-4 and M9T337 by treating them with a 75 mM NaCl solution for 144 h to see how they handled the salt stress. ZM-4 was found to be more tolerant to salt stress when tested using fresh weight. The tolerance of leaves and roots differed significantly. The tolerance of the leaves of ZM-4 was primarily due to their higher content of flavonoids, and they had a strong antioxidant capacity. Not only did the ZM-4 roots have a greater anti-oxidant capacity, but they also contained more osmotic regulators such as amino acids and sugars (L-proline, tran-4-hydroxy-L-prolin, D−fructose 6−phosphate, D−glucose 6−phosphate, D−sorbitol, and so on) under normal conditions, and even more amino acids and sugars (N-methyl-trans-4-hydroxy-L-proline, 2-hydroxyethylphosphonic acid, D-sucrose, maltotriose, melezitose, and so on) induced by salt stress. Furthermore, the genes (*GLT1*, *ALDC*, *AA5GT*, *BAM7*, *CM3*, *METK4*, *PKP1*, *AMY1.1*, etc.) that responded in both directions were highly expressed. This research could serve as a theoretical foundation for the use of salt-tolerant ZM-4 in the breeding of salt-tolerant apple rootstocks.

## Data Availability

The raw sequence data reported in this paper have been deposited in the Genome Sequence Archive (Genomics, Proteomics & Bioinformatics 2021) [77] in the National Genomics Data Center (Nucleic Acids Res 2022) [78], China National Center for Bioinformation/Beijing Institute of Genomics, Chinese Academy of Sciences (GSA: CRA009194, accessed on 10 December 2022), which is publicly accessible at https://ngdc.cncb.ac.cn/gsa.

## Supplementary Materials

The following supporting information can be downloaded at: https://www.mdpi.com/article/10.3390/ijms24043638/s1.
