# Supplementary material for: Transcriptome and Metabolome Analysis Reveals Salt-Tolerance Pathways in the Leaves and Roots of ZM-4 (Malus zumi) in the Early Stages of Salt Stress
Source: Int J Mol Sci. 2023 Feb 11;24(4):3638. doi: 10.3390/ijms24043638 (PMC9960305; doi:10.3390/ijms24043638)
Supplement: Supplementary file 1 [file ijms-24-03638-s001.zip › ijms-2193630-supplementary/Supplementary Figures.docx]

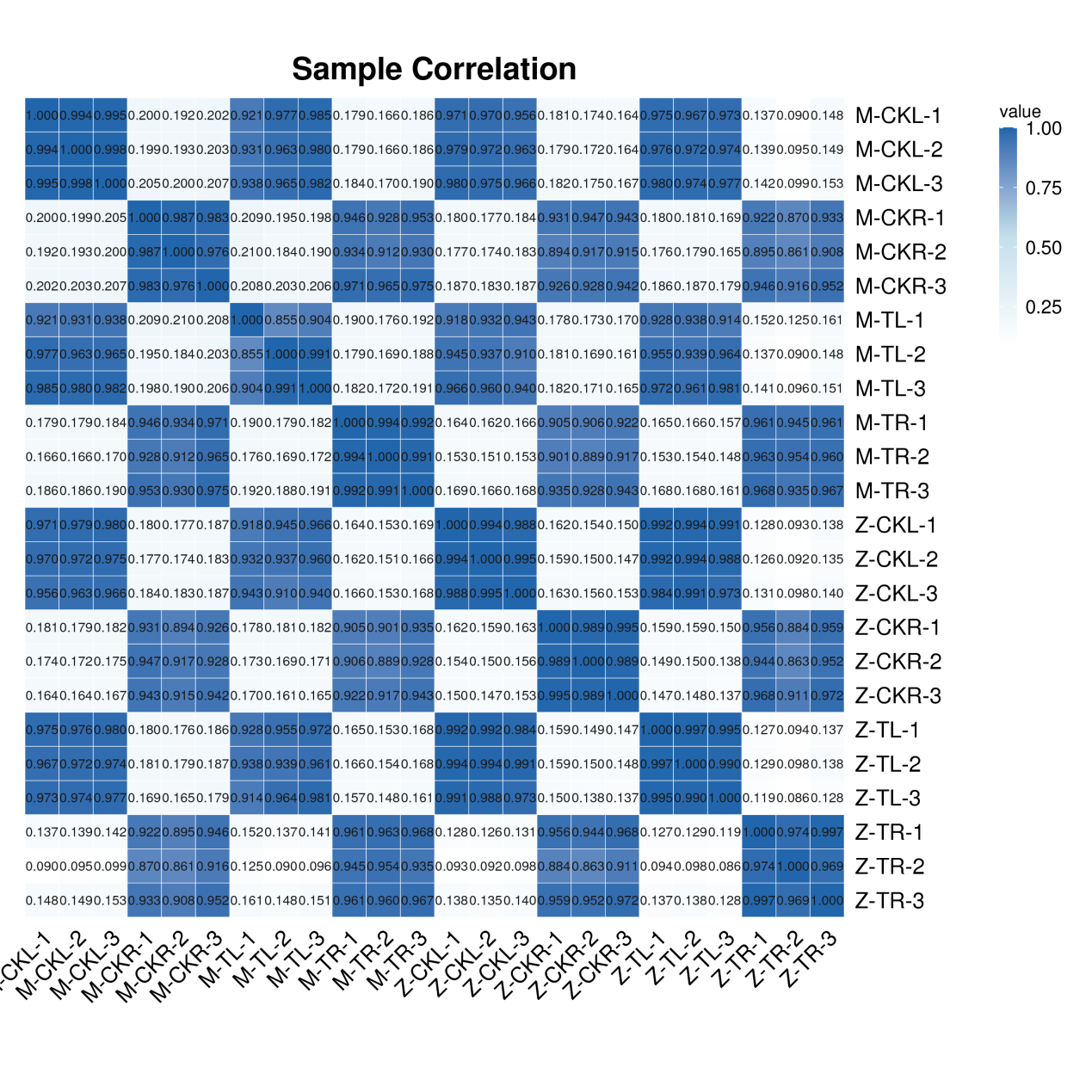


**Figure S1** Heatmap of pearson’s correlation coefficient between replicates of samples

**
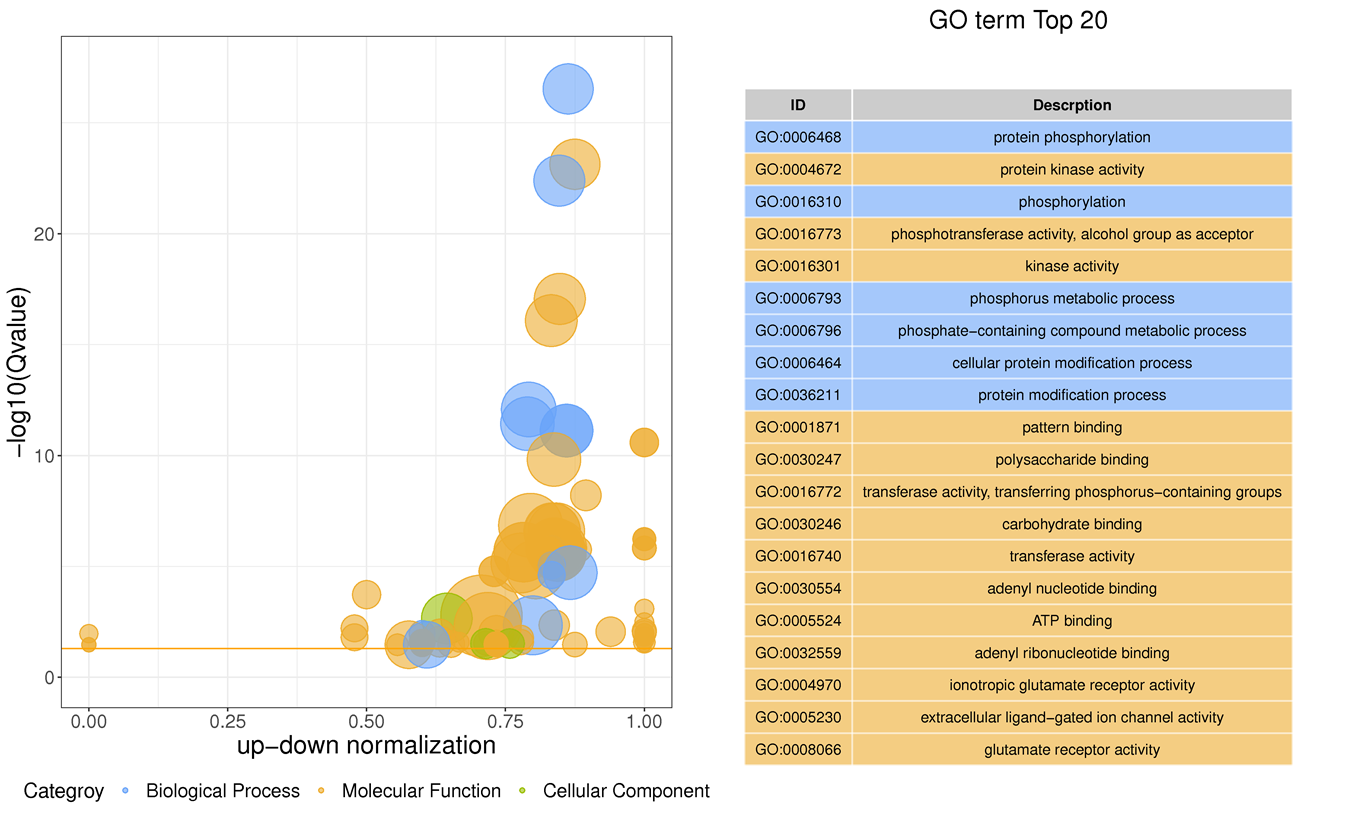
**

**Figure S2** The top 20 GO terms of DEGs in M-CKL vs M-TL


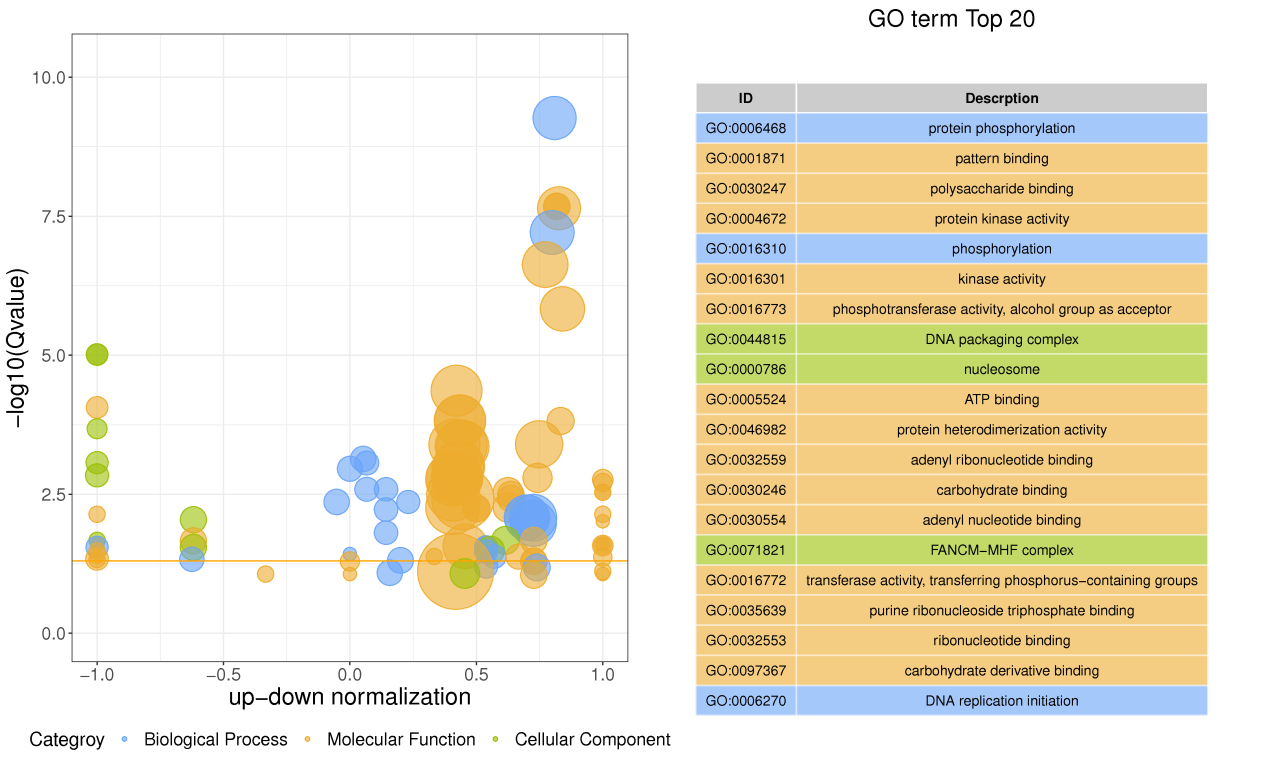


**Figure S3** The top 20 GO terms of DEGs in Z-CKL vs Z-TL


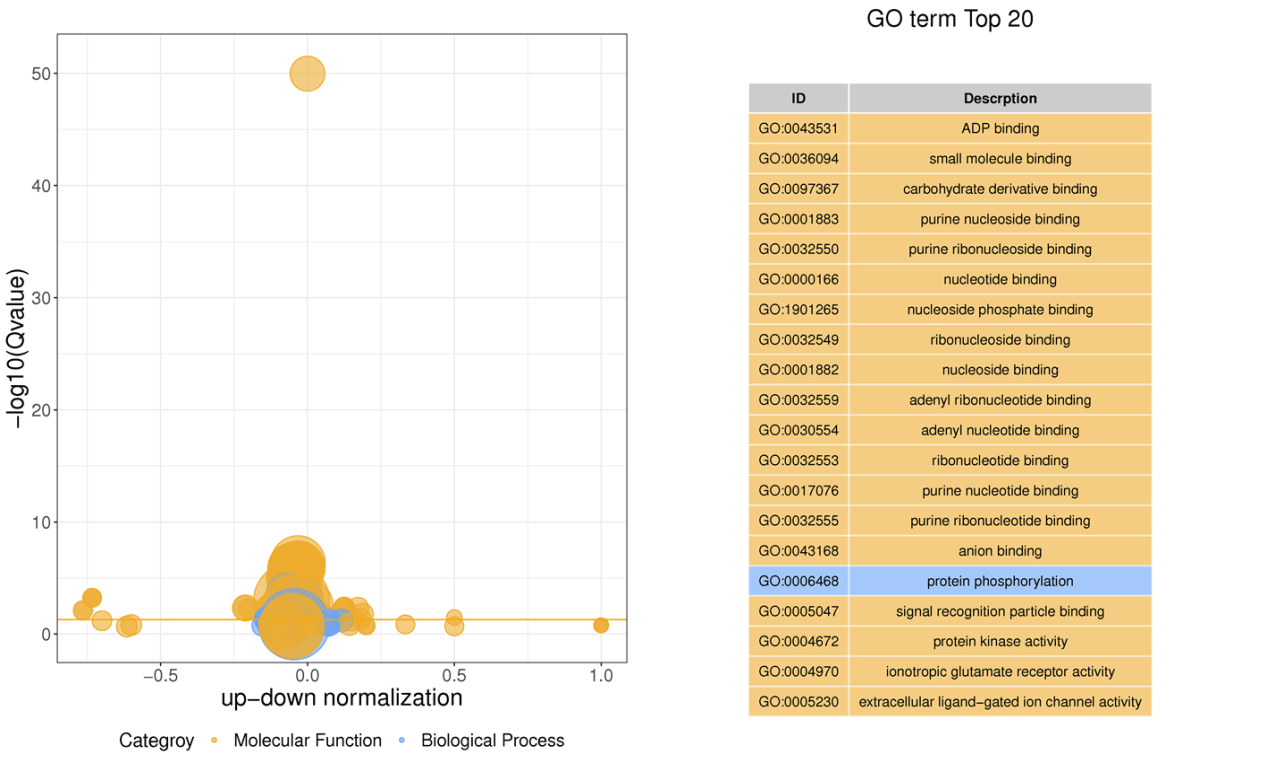


**Figure S4** The top 20 GO terms of DEGs in M-TL vs Z-TL


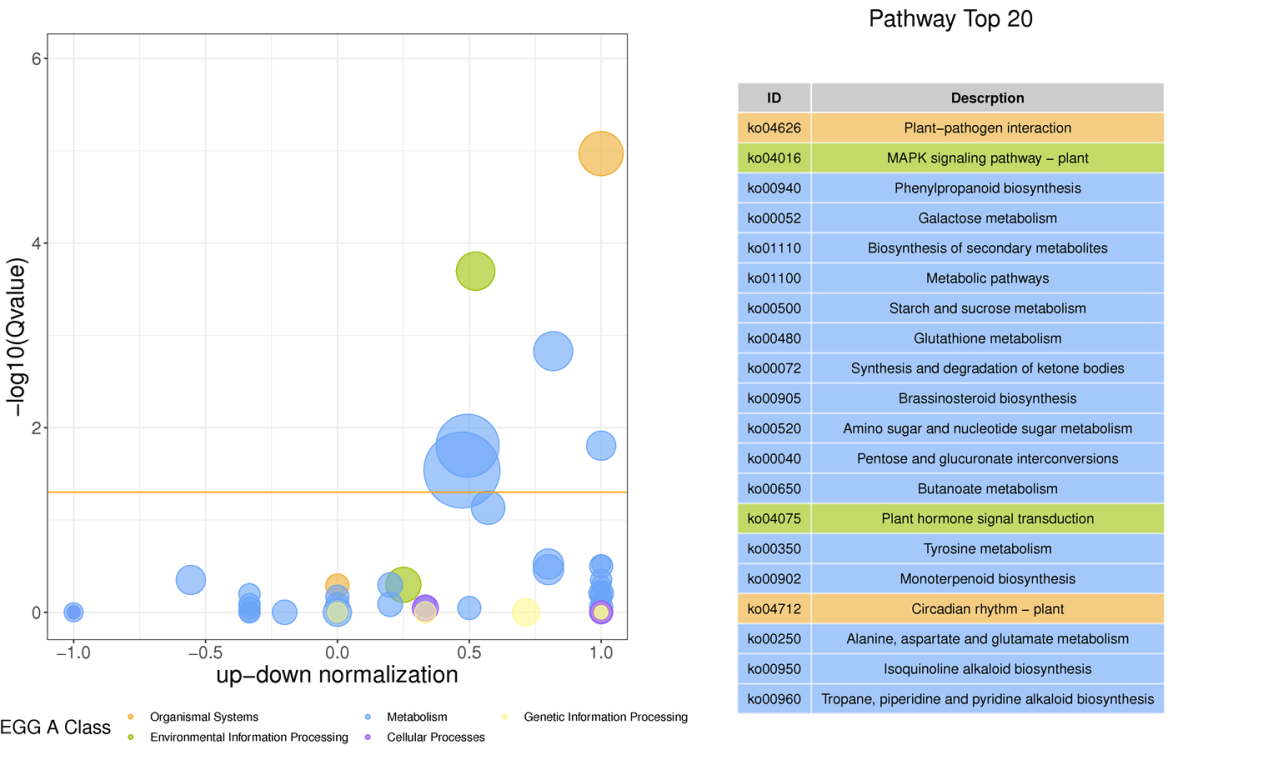


**Figure S5** The top 20 KEGG pathways of DEGs in M-CKL vs M-TL


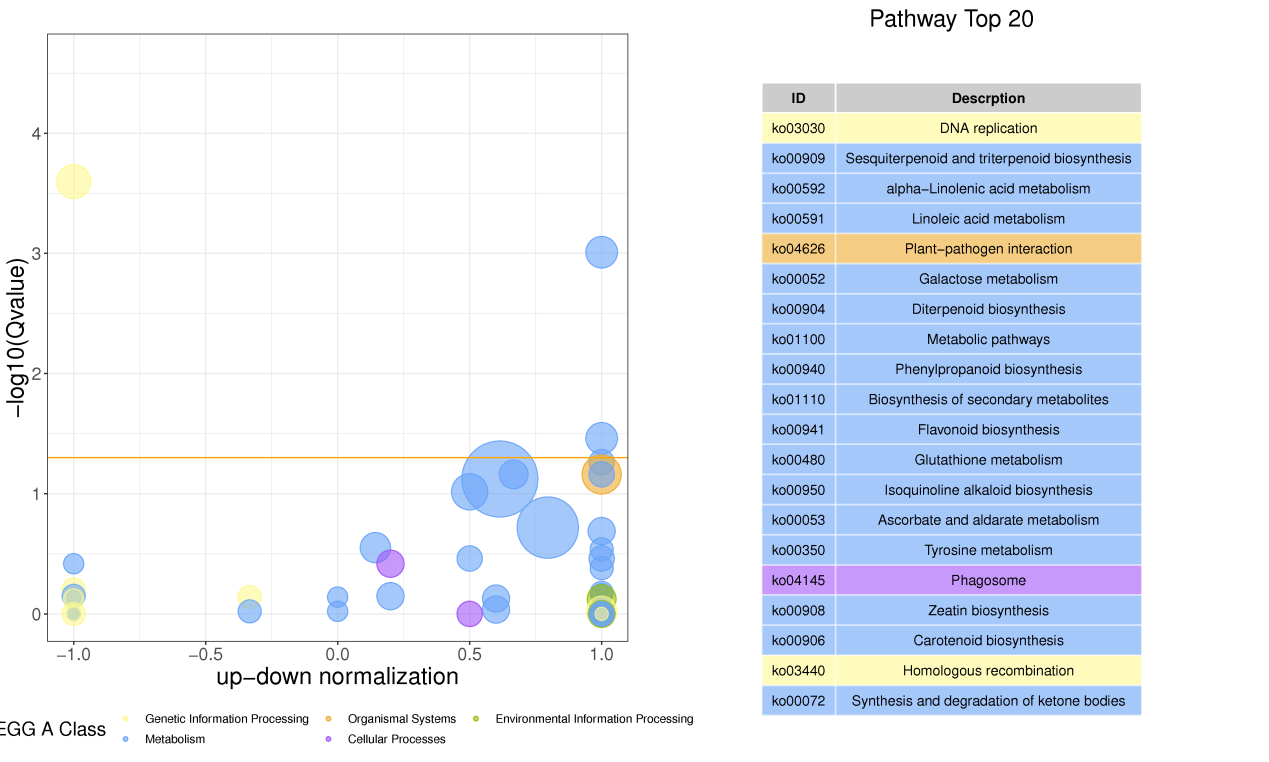


**Figure S6** The top 20 KEGG pathways of DEGs in Z-CKL vs Z-TL


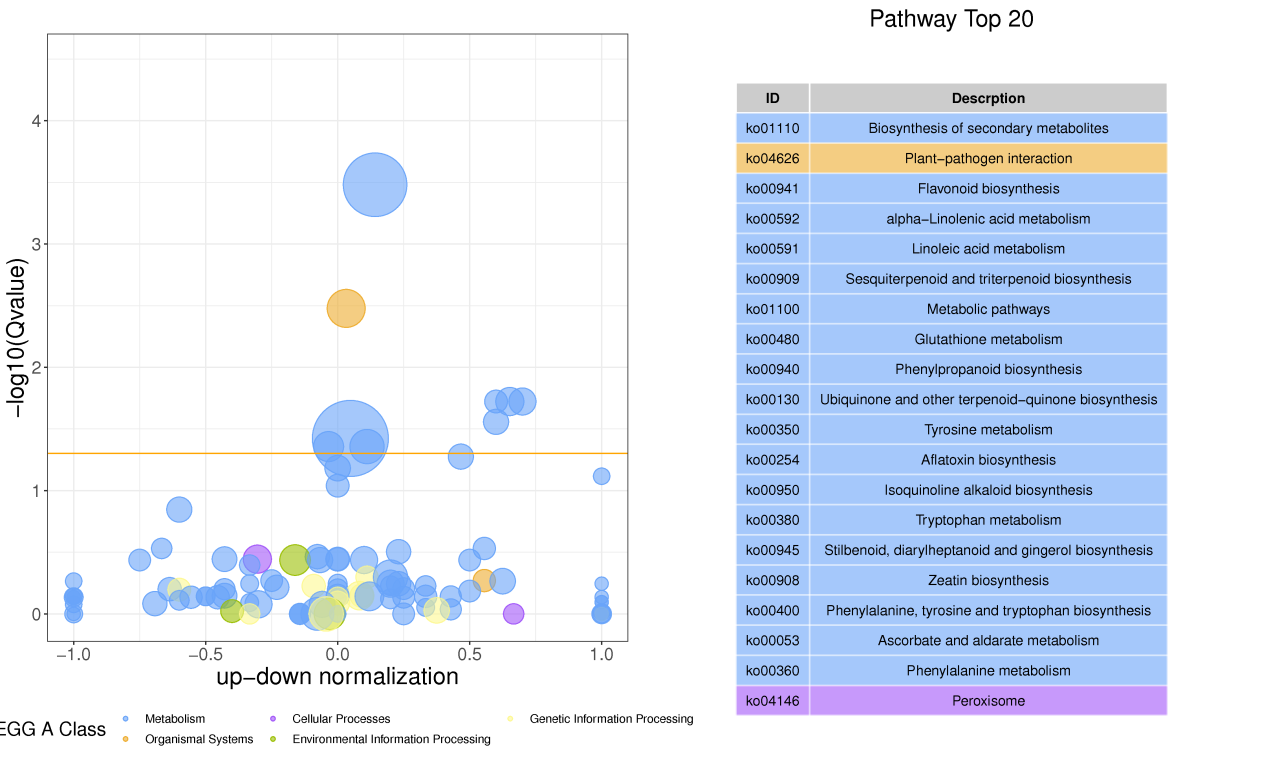


**Figure S7** The top 20 KEGG pathways of DEGs in M-TL vs Z-TL


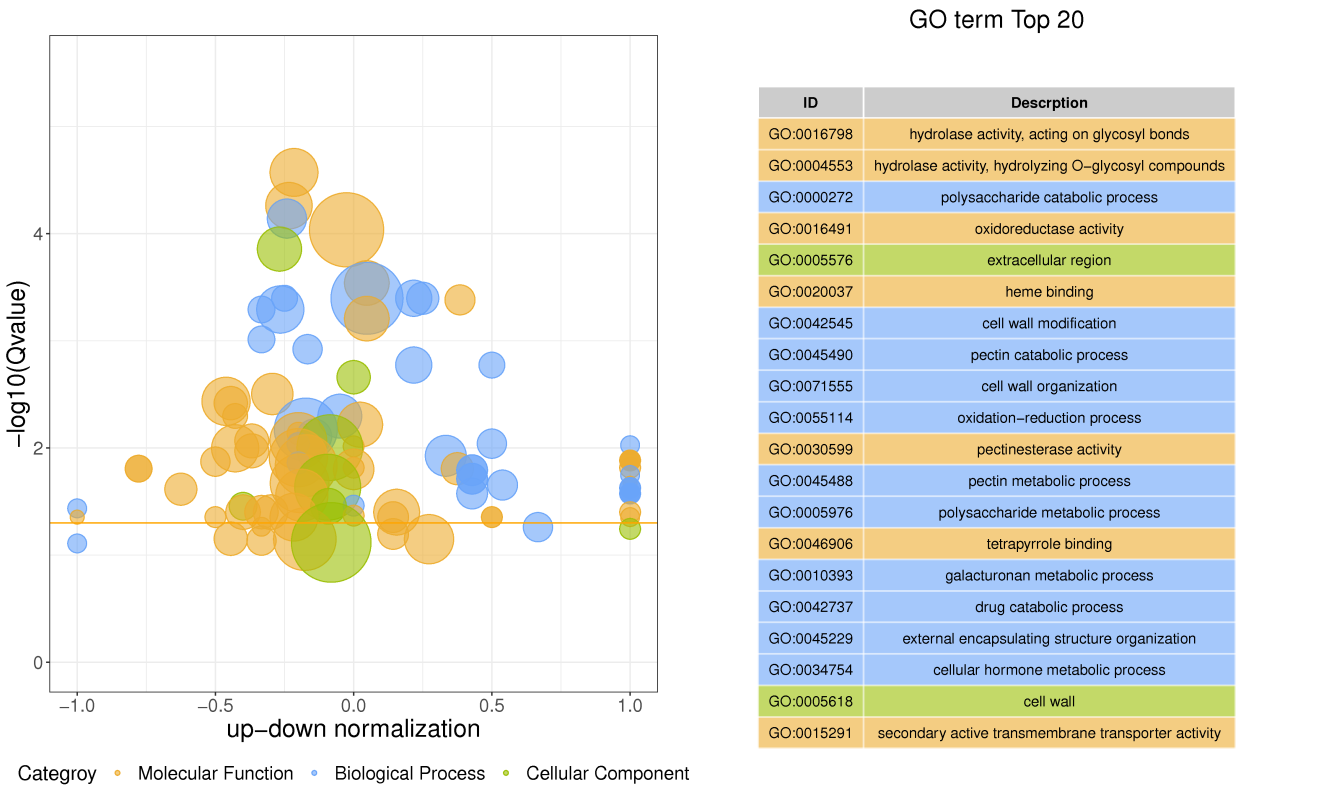


**Figure S8** The top 20 GO terms of DEGs in M-CKR vs M-TR


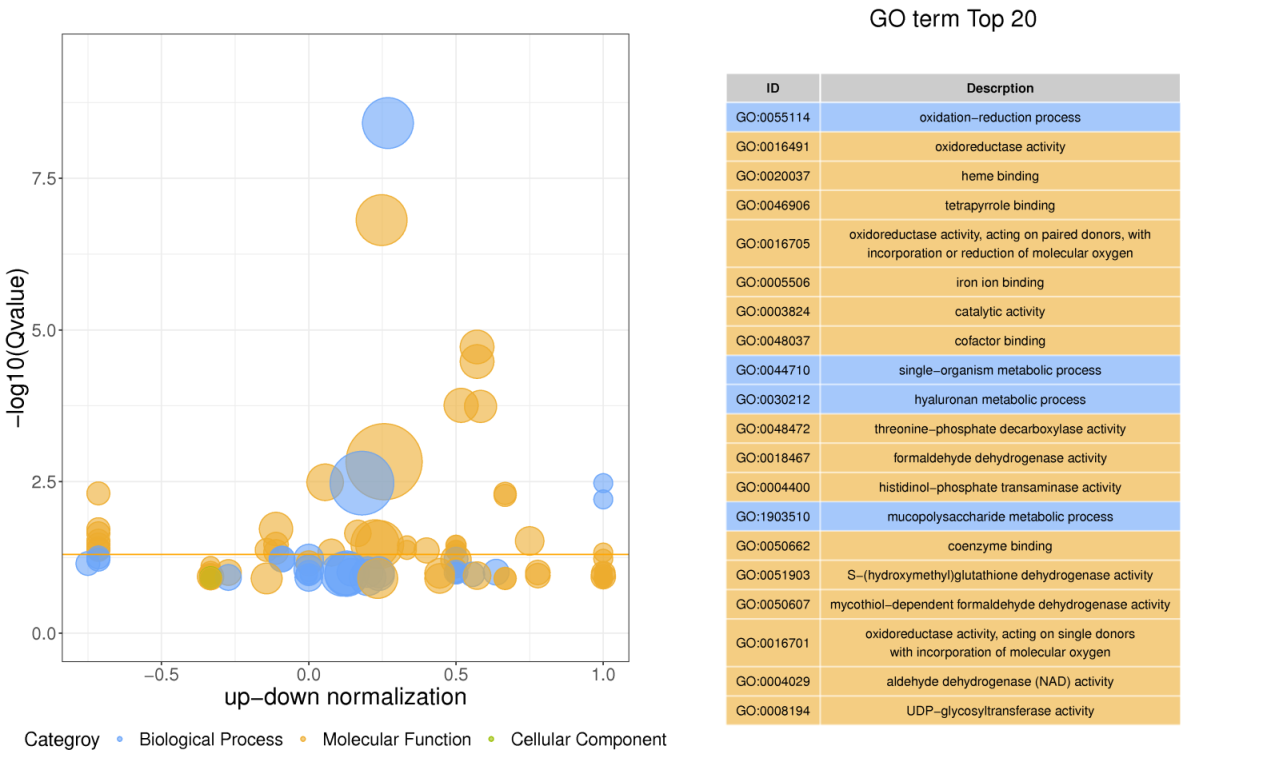


**Figure S9** The top 20 GO terms of DEGs in Z-CKR vs Z-TR


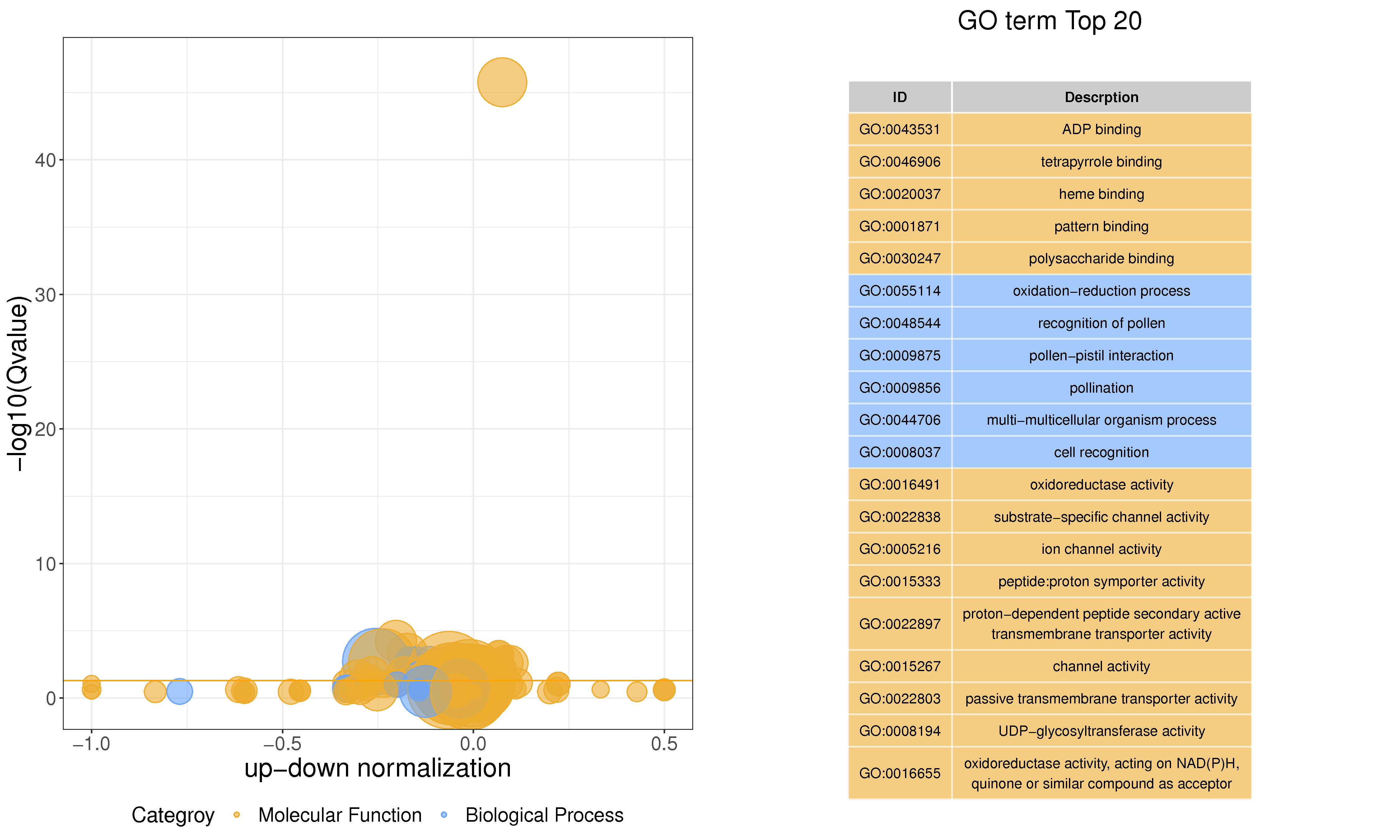


**Figure S10** The top 20 GO terms of DEGs in M-TR vs Z-TR


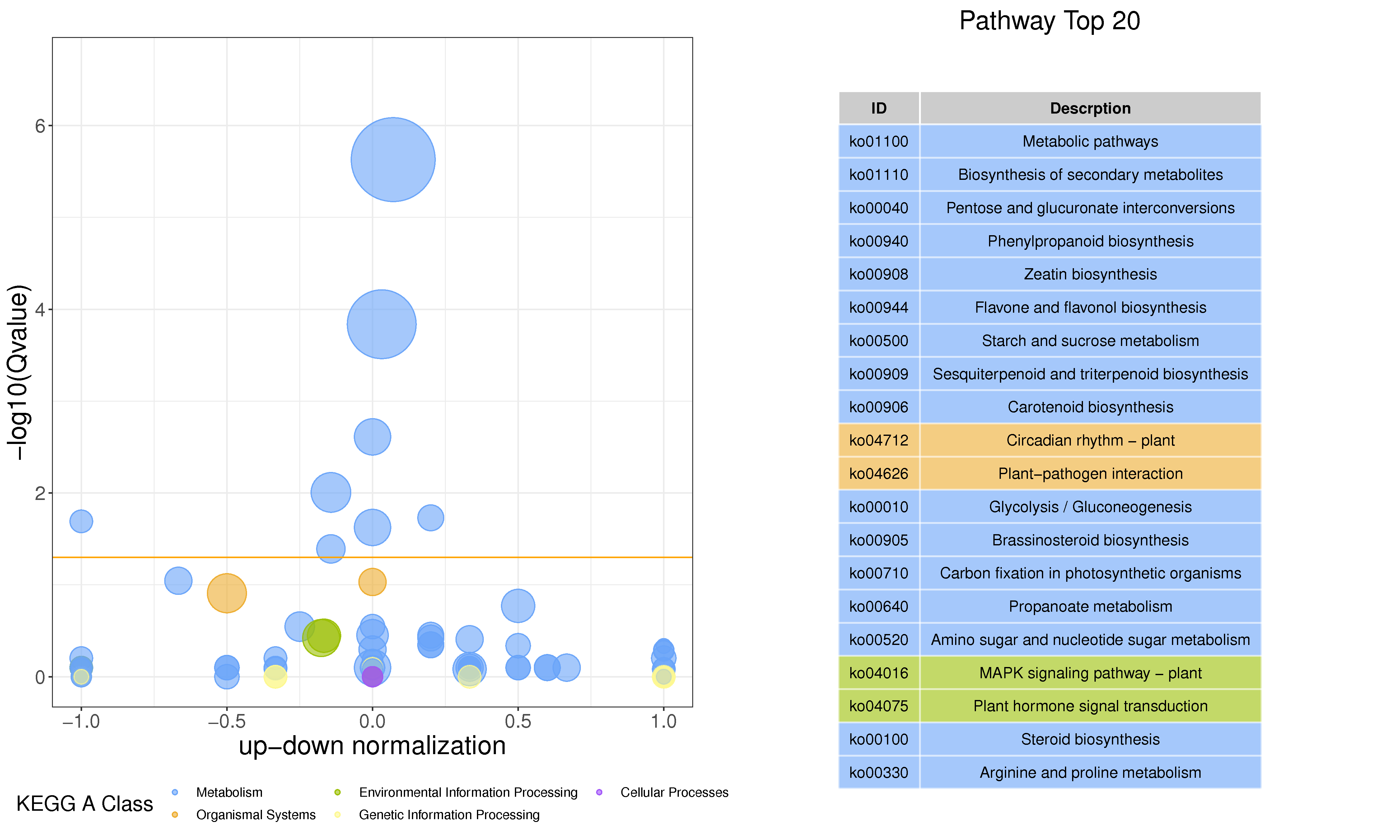


**Figure S11** The top 20 KEGG pathways of DEGs in M-CKR vs M-TR


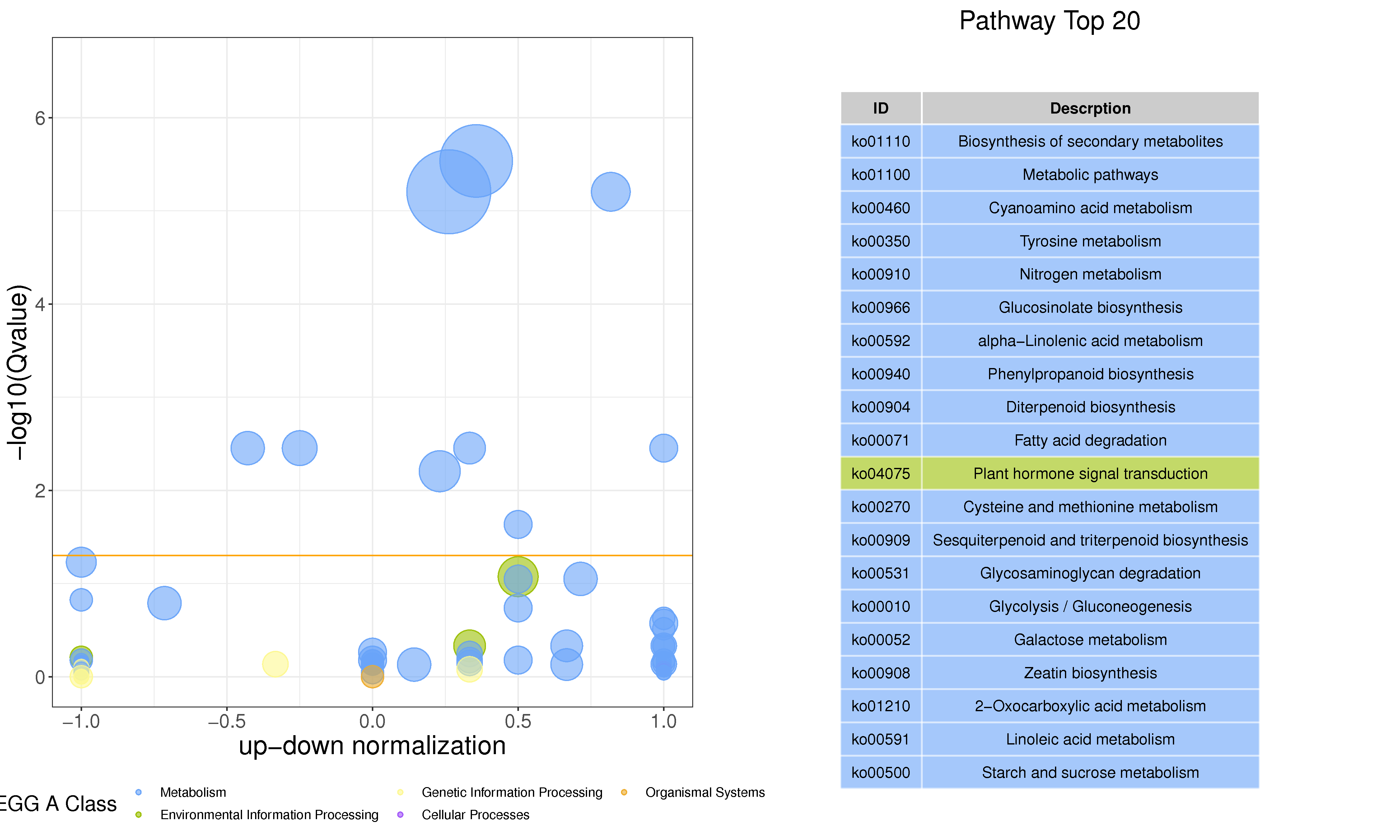


**Figure S12** The top 20 KEGG pathways of DEGs in Z-CKR vs Z-TR


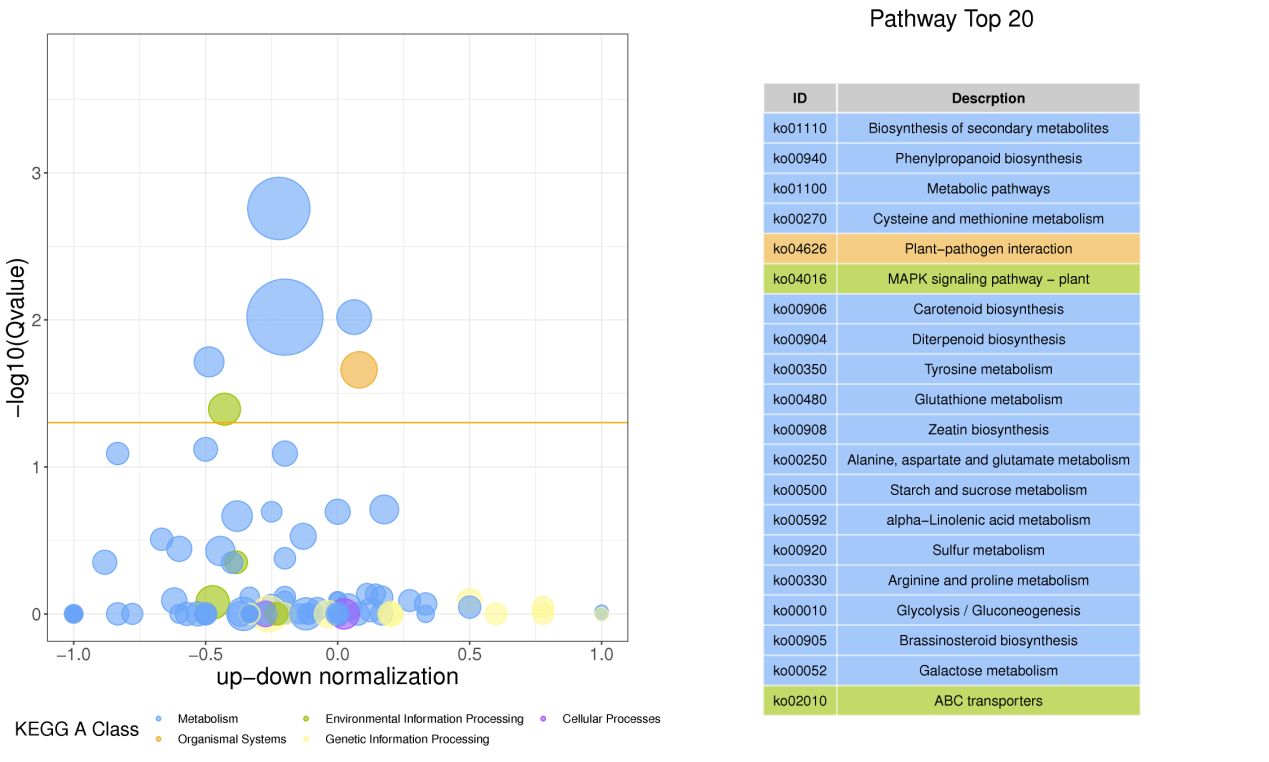


**Figure S13** The top 20 KEGG pathways of DEGs in M-TR vs Z-TR


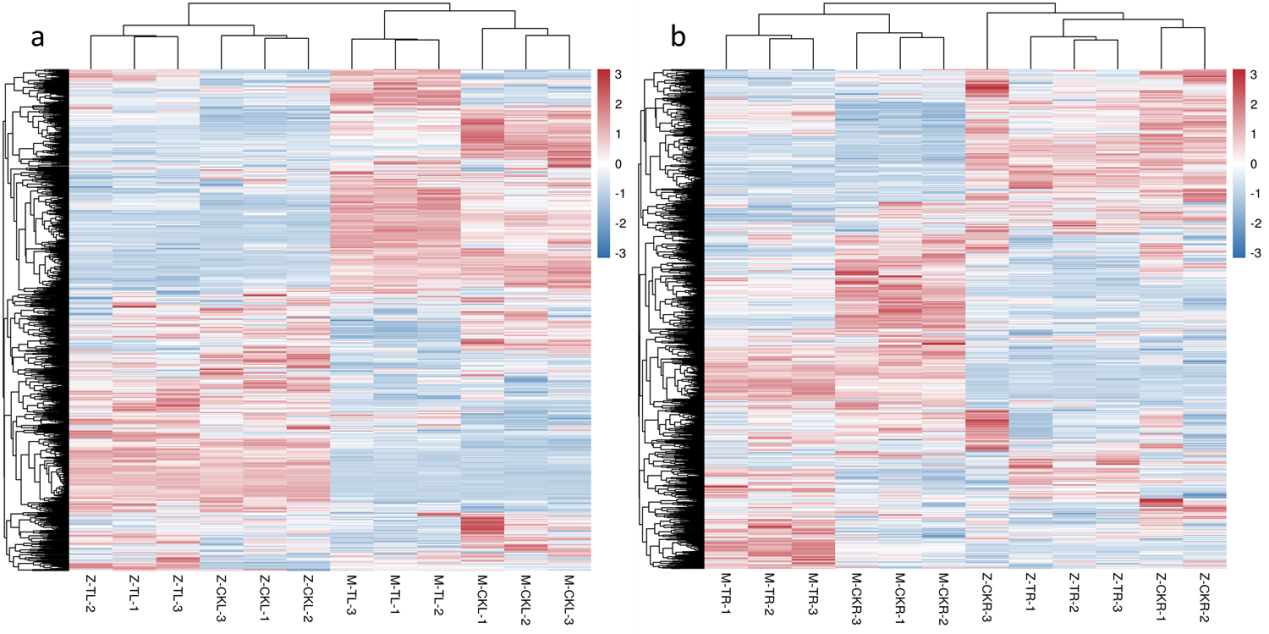


**Figure S14** Heatmap of metabolites of leaves and roots in ZM-4 and M9T337 under salt stress. (a) Heatmap of metabolites of leaves in ZM-4 and M9T337 under salt stress. (b) Heatmap of metabolites of roots in ZM-4 and M9T337 under salt stress.


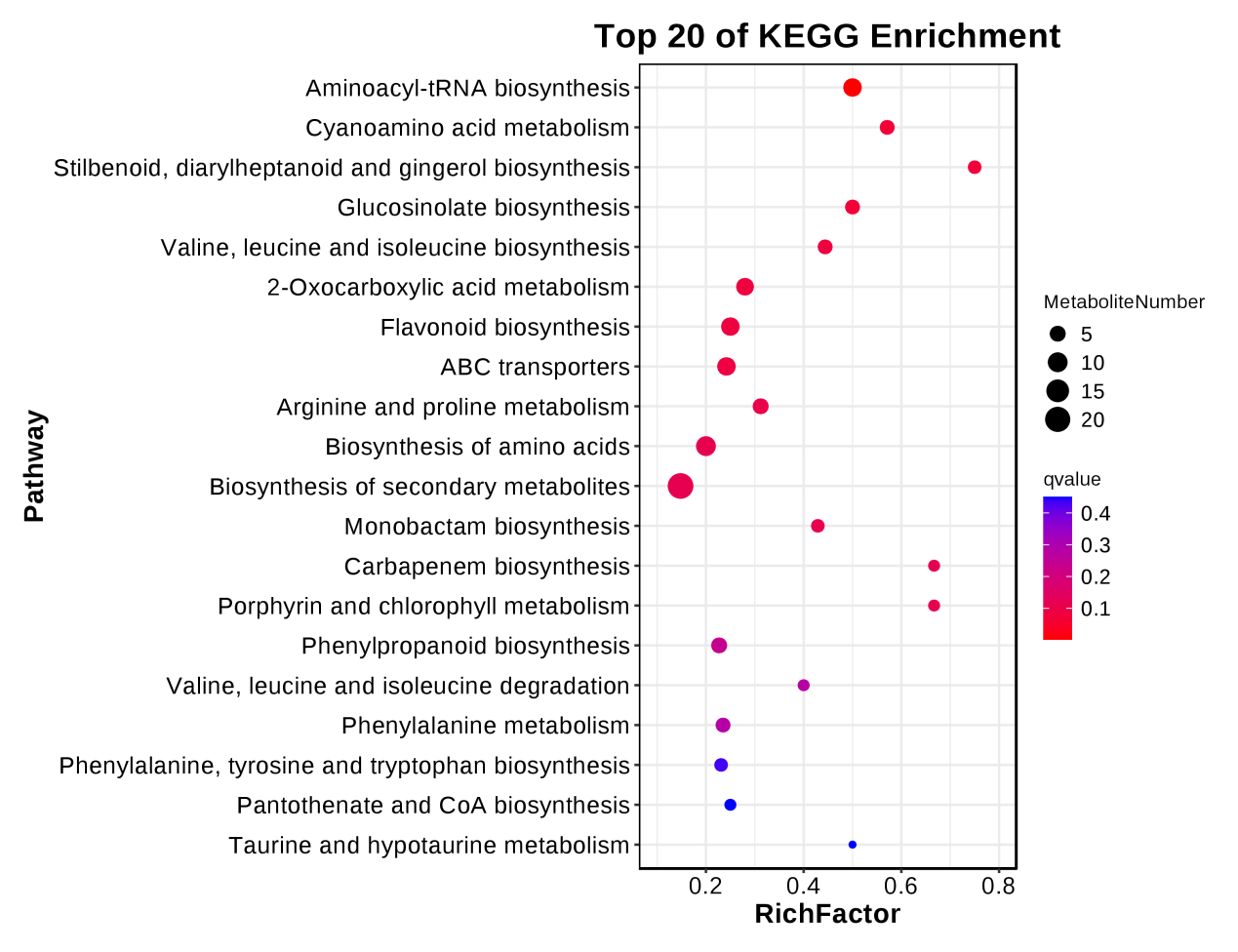


**Figure S15** The top 20 KEGG pathways of DAMs in M-CKL vs M-TL


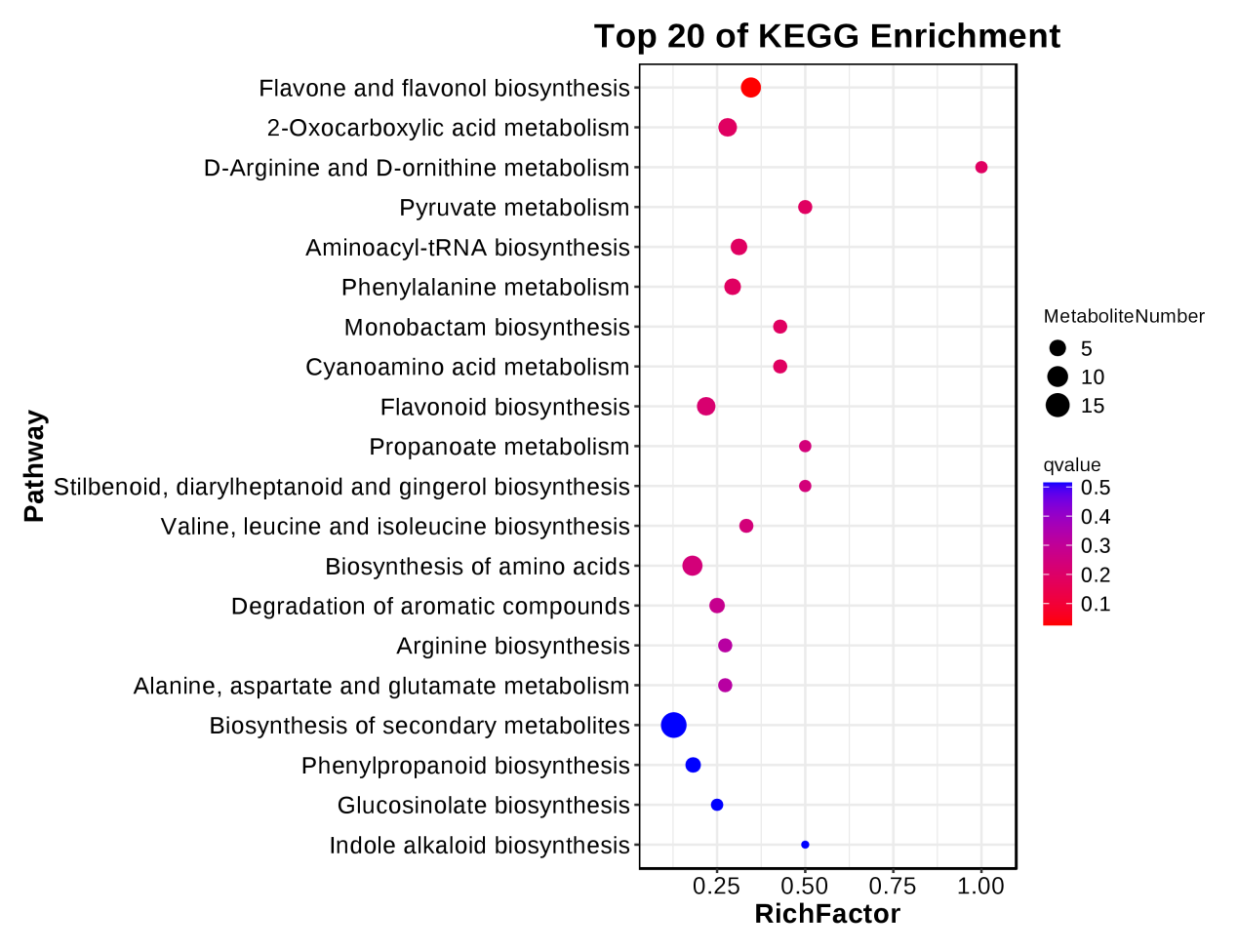


**Figure S16** The top 20 KEGG pathways of DAMs in Z-CKL vs Z-TL


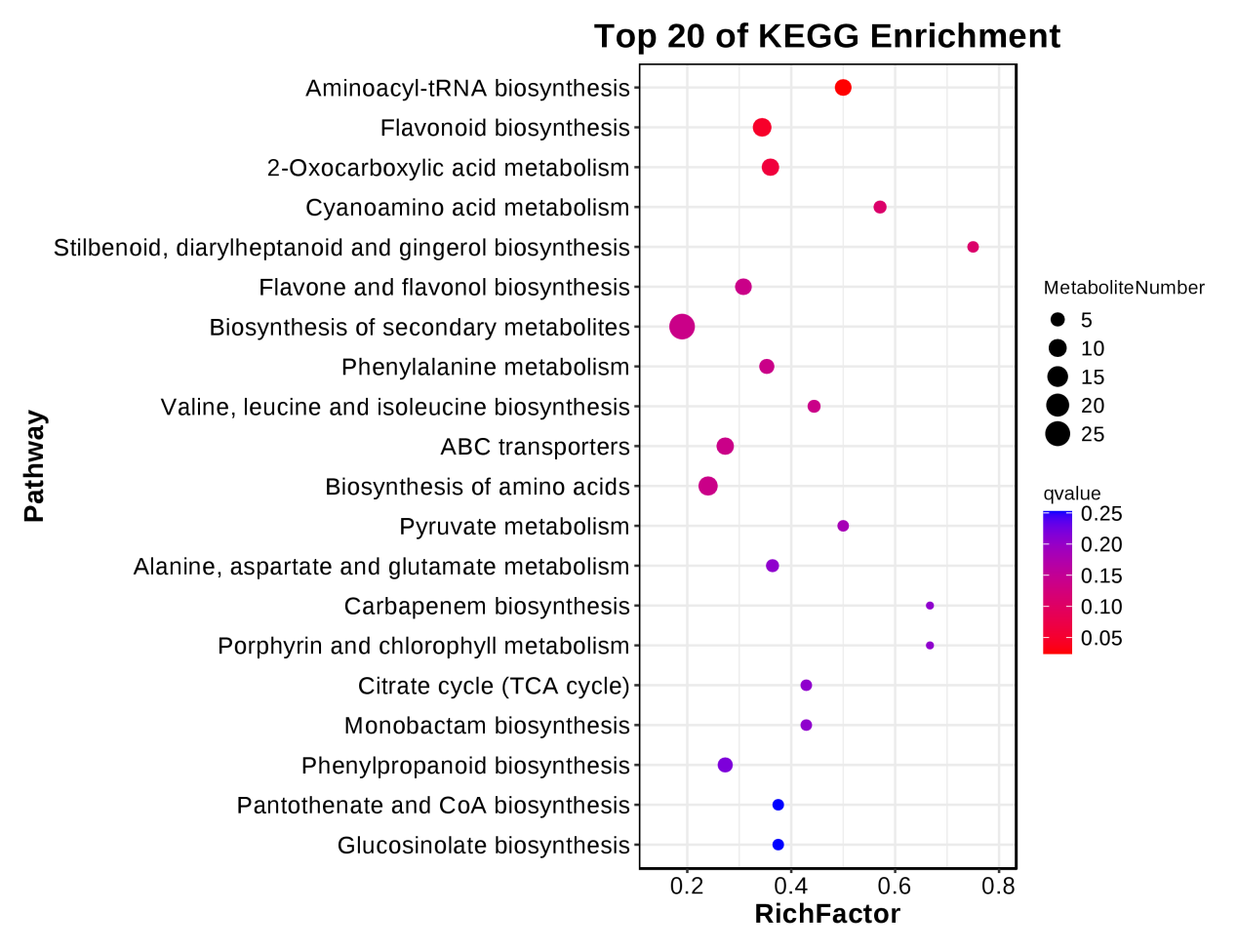


**Figure S17** The top 20 KEGG pathways of DAMs in M-TL vs Z-TL


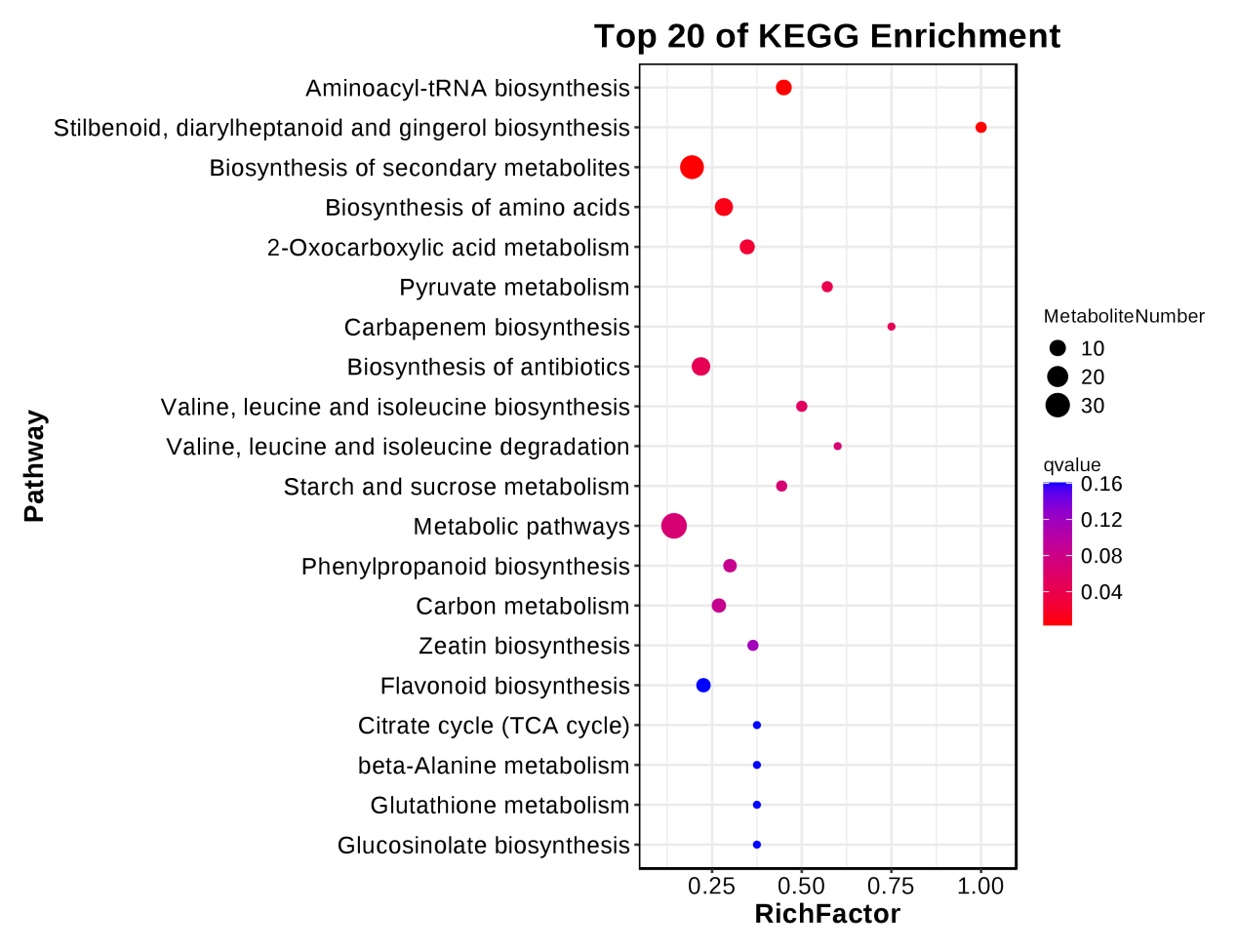


**Figure S18** The top 20 KEGG pathways of DAMs in M-CKR vs M-TR


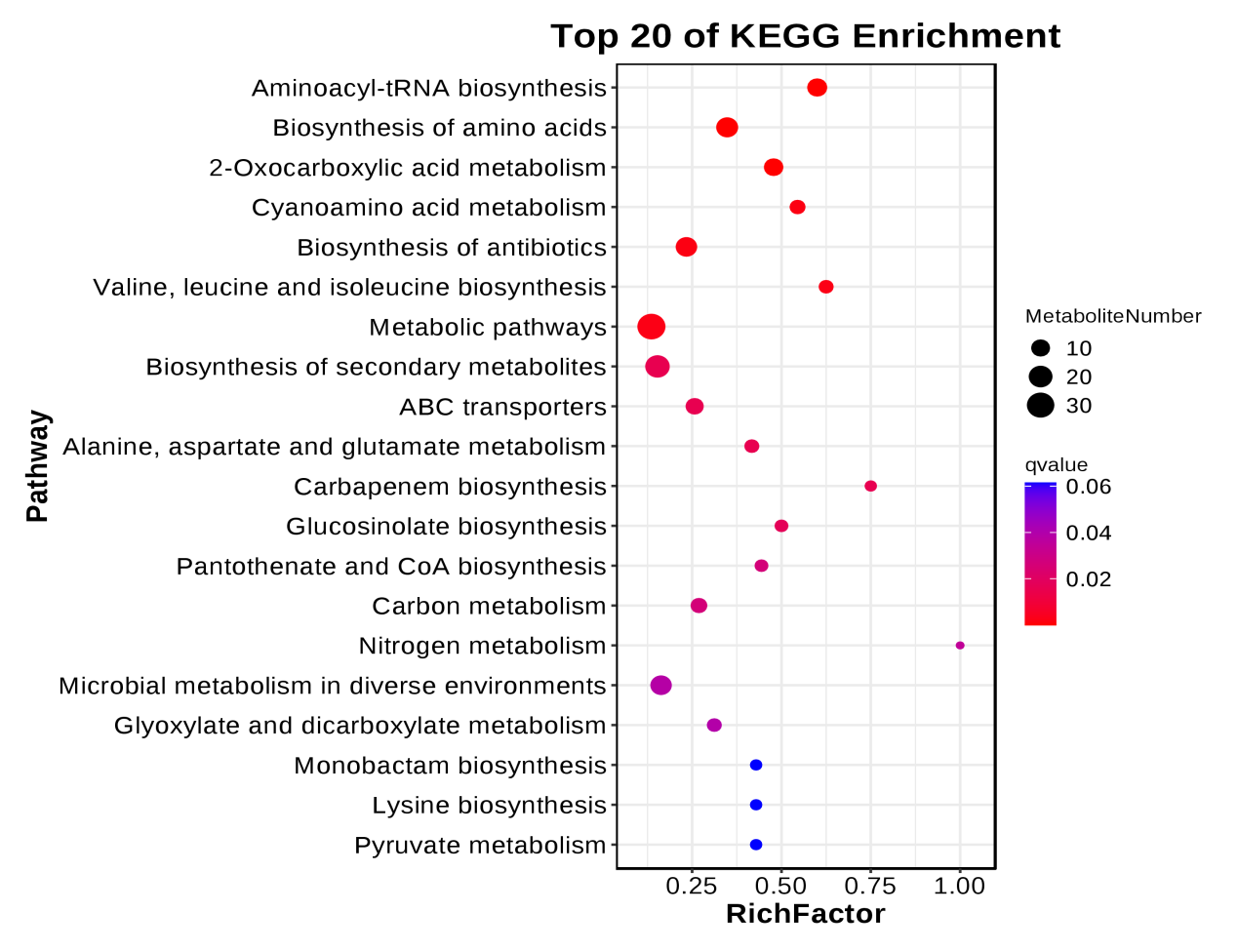


**Figure S19** The top 20 KEGG pathways of DAMs in Z-CKR vs Z-TR


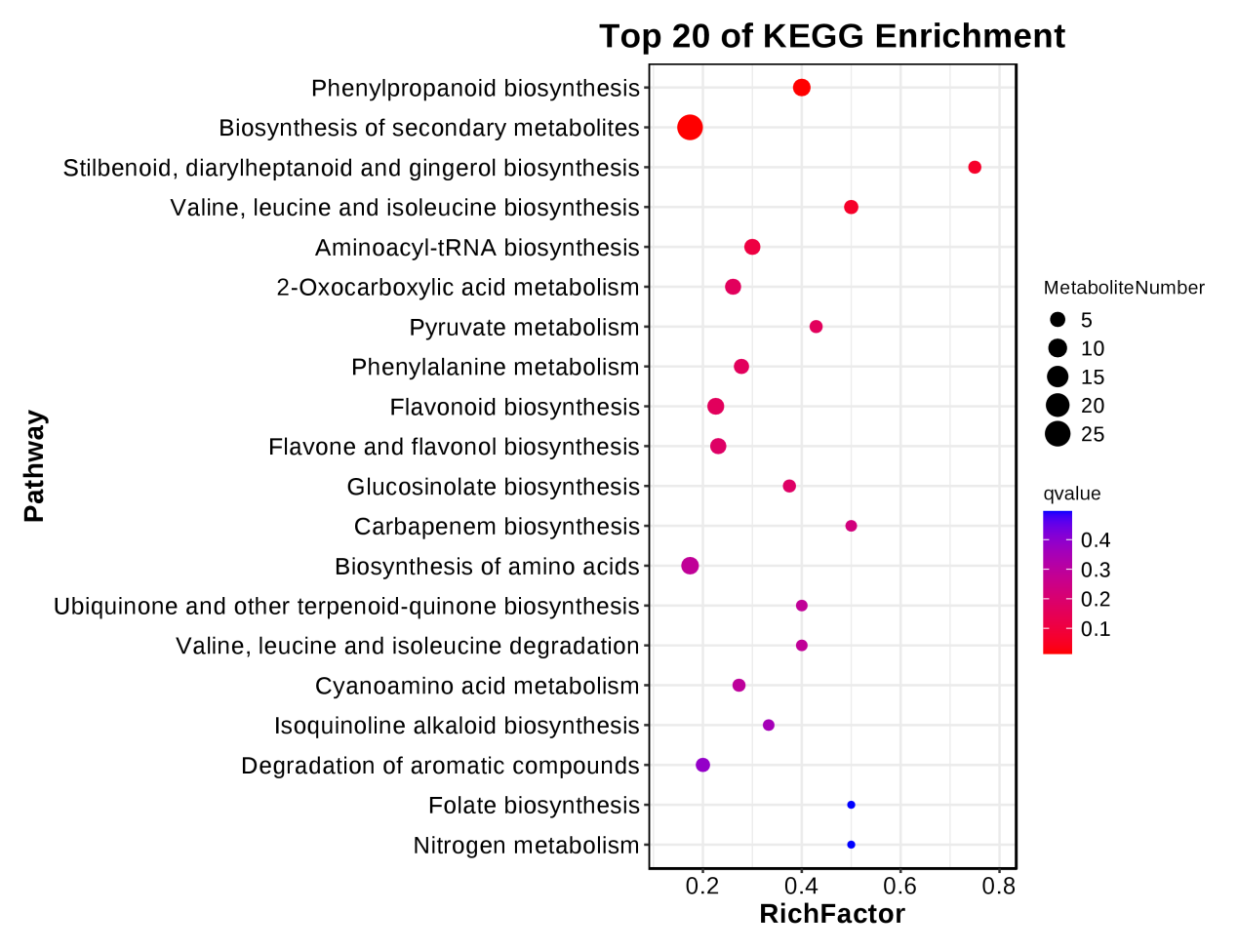


**Figure S20** The top 20 KEGG pathways of DAMs in M-TR vs Z-TR


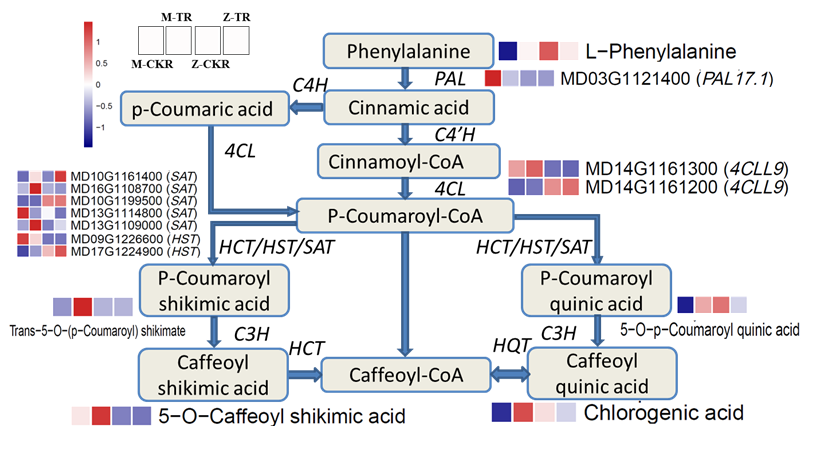


**Figure S21** The main salt resistance genes and metabolites in phenylpropanoid biosynthesis pathway (PAL, phenylalanine ammonia-lyase; C4’H, cinnamate-4’-hydroxylase; C4H, cinnamate-4-hydroxylase; 4CL, 4-Coumarate:coenzyme A ligase; HCT/HAT/SAT, Shikimate/Quinate Hydroxycinnamoyltransferase; C3H,p-coumaroyl shikimate 3-hydroxylase; HQT, hydroxycinnamoyl CoA quinate hydrocycinnamoyl transferase).
