# Supplementary material for: Transcriptome and Metabolome Analysis Reveals Salt-Tolerance Pathways in the Leaves and Roots of ZM-4 (Malus zumi) in the Early Stages of Salt Stress
Source: Int J Mol Sci. 2023 Feb 11;24(4):3638. doi: 10.3390/ijms24043638 (PMC9960305; doi:10.3390/ijms24043638)
Supplement: Supplementary file 1 [file ijms-24-03638-s001.zip › ijms-2193630-supplementary/Supplementary Figures.pdf]

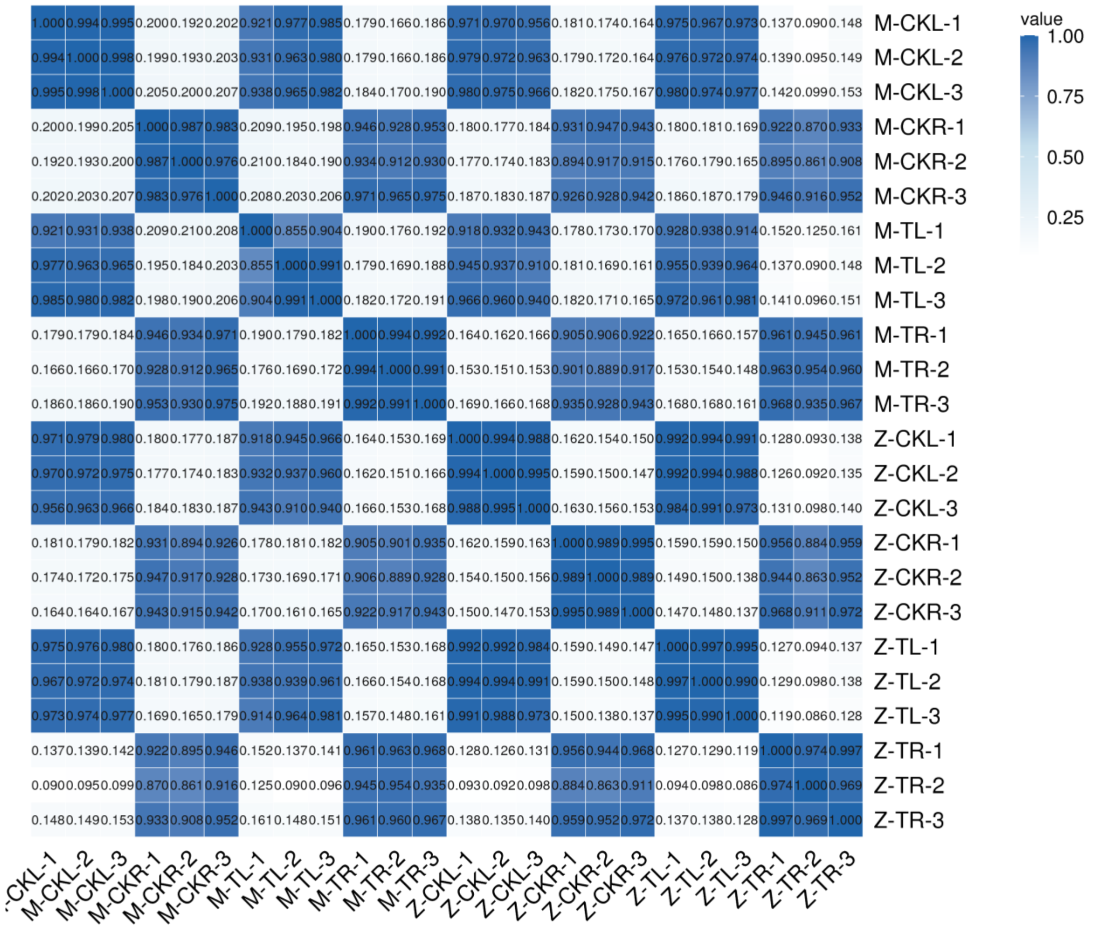

Figure S1 Heatmap of pearson's correlation coefficient between replicates of samples

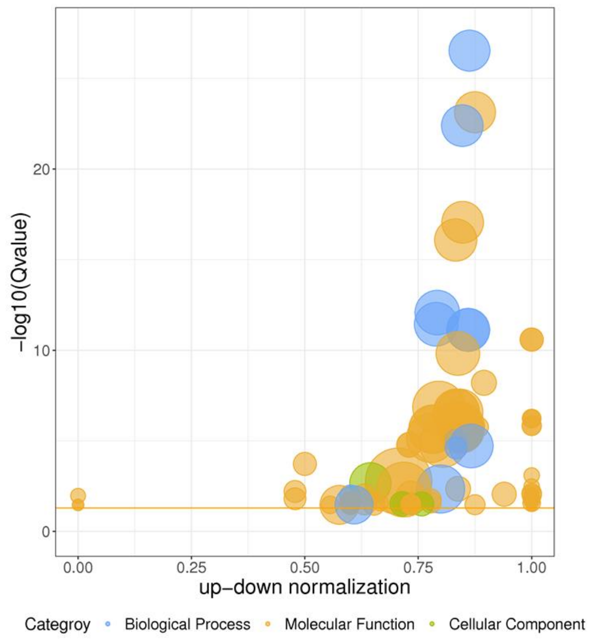

GO term Top 20

ID	Descrption
GO:0006468	protein phosphorylation
GO:0004672	protein kinase activity
GO:0016310	phosphorylation
GO:0016773	phosphotransferase activity, alcohol group as acceptor
GO:0016301	kinase activity
GO:0006793	phosphorus metabolic process
GO:0006796	phosphate−containing compound metabolic process
GO:0006464	cellular protein modification process
GO:0036211	protein modification process
GO:0001871	pattern binding
GO:0030247	polysaccharide binding
GO:0016772	transferase activity, transferring phosphorus−containing groups
GO:0030246	carbohydrate binding
GO:0016740	transferase activity
GO:0030554	adenyl nucleotide binding
GO:0005524	ATP binding
GO:0032559	adenyl ribonucleotide binding
GO:0004970	ionotropic glutamate receptor activity
GO:0005230	extracellular ligand−gated ion channel activity
GO:0008066	glutamate receptor activity

Figure S2 The top 20 GO terms of DEGs in M-CKL vs M-TL

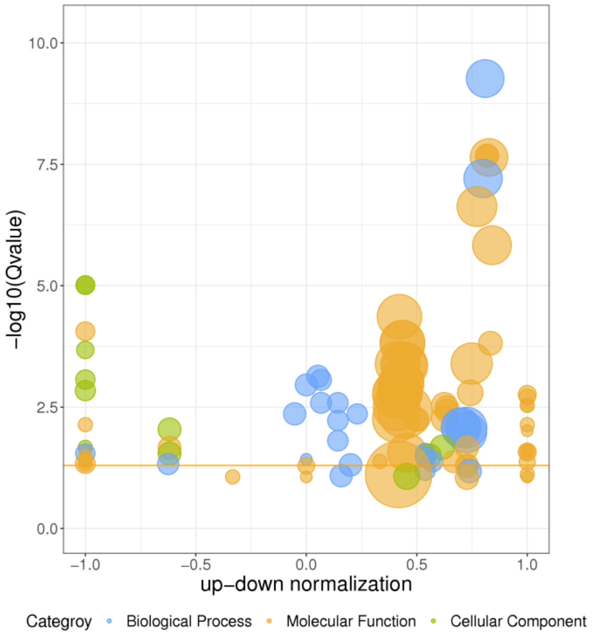

GO term Top 20

ID	Descrption
GO:0006468	protein phosphorylation
GO:0001871	pattern binding
GO:0030247	polysaccharide binding
GO:0004672	protein kinase activity
GO:0016310	phosphorylation
GO:0016301	kinase activity
GO:0016773	phosphotransferase activity, alcohol group as acceptor
GO:0044815	DNA packaging complex
GO:0000786	nucleosome
GO:0005524	ATP binding
GO:0046982	protein heterodimerization activity
GO:0032559	adenyl ribonucleotide binding
GO:0030246	carbohydrate binding
GO:0030554	adenyl nucleotide binding
GO:0071821	FANCM−MHF complex
GO:0016772	transferase activity, transferring phosphorus−containing groups
GO:0035639	purine ribonucleoside triphosphate binding
GO:0032553	ribonucleotide binding
GO:0097367	carbohydrate derivative binding
GO:0006270	DNA replication initiation

Figure S3 The top 20 GO terms of DEGs in Z-CKL vs Z-TL

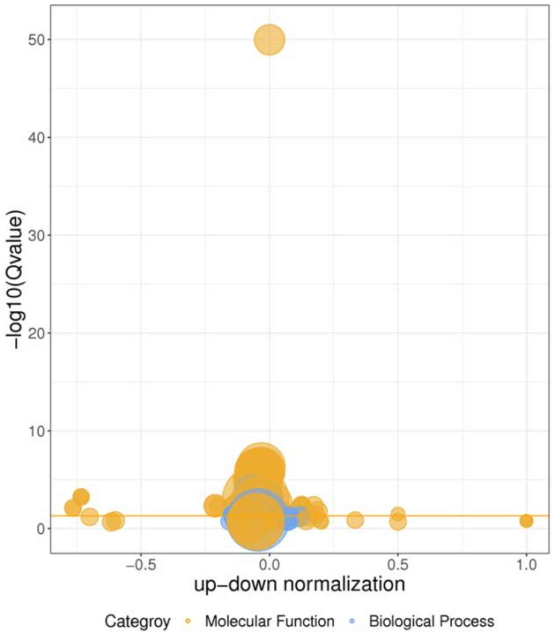

GO term Top 20

ID	Descrption
GO:0043531	ADP binding
GO:0036094	small molecule binding
GO:0097367	carbohydrate derivative binding
GO:0001883	purine nucleoside binding
GO:0032550	purine ribonucleoside binding
GO:0000166	nucleotide binding
GO:1901265	nucleoside phosphate binding
GO:0032549	ribonucleoside binding
GO:0001882	nucleoside binding
GO:0032559	adenyl ribonucleotide binding
GO:0030554	adenyl nucleotide binding
GO:0032553	ribonucleotide binding
GO:0017076	purine nucleotide binding
GO:0032555	purine ribonucleotide binding
GO:0043168	anion binding
GO:0006468	protein phosphorylation
GO:0005047	signal recognition particle binding
GO:0004672	protein kinase activity
GO:0004970	ionotropic glutamate receptor activity
GO:0005230	extracellular ligand−gated ion channel activity

Figure S4 The top 20 GO terms of DEGs in M-TL vs Z-TL

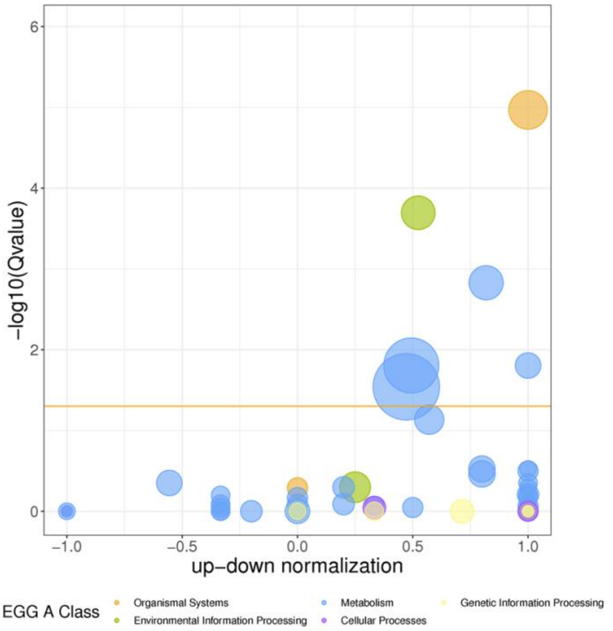

Pathway Top 20

ID	Descrption
ko04626	Plant−pathogen interaction
ko04016	MAPK signaling pathway – plant
ko00940	Phenylpropanoid biosynthesis
ko00052	Galactose metabolism
ko01110	Biosynthesis of secondary metabolites
ko01100	Metabolic pathways
ko00500	Starch and sucrose metabolism
ko00480	Glutathione metabolism
ko00072	Synthesis and degradation of ketone bodies
ko00905	Brassinosteroid biosynthesis
ko00520	Amino sugar and nucleotide sugar metabolism
ko00040	Pentose and glucuronate interconversions
ko00650	Butanoate metabolism
ko04075	Plant hormone signal transduction
ko00350	Tyrosine metabolism
ko00902	Monoterpenoid biosynthesis
ko04712	Circadian rhythm – plant
ko00250	Alanine, aspartate and glutamate metabolism
ko00950	Isoquinoline alkaloid biosynthesis
ko00960	Tropane, piperidine and pyridine alkaloid biosynthesis

Figure S5 The top 20 KEGG pathways of DEGs in M-CKL vs M-TL

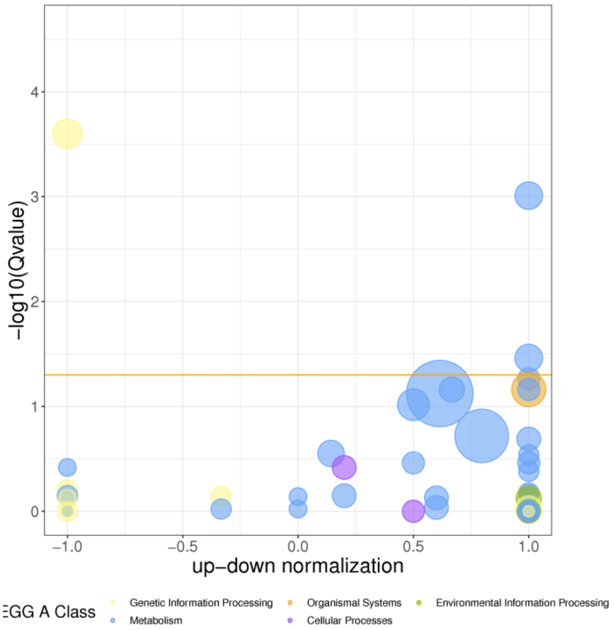

Pathway Top 20

ID	Descrption
ko03030	DNA replication
ko00909	Sesquiterpenoid and triterpenoid biosynthesis
ko00592	alpha−Linolenic acid metabolism
ko00591	Linoleic acid metabolism
ko04626	Plant−pathogen interaction
ko00052	Galactose metabolism
ko00904	Diterpenoid biosynthesis
ko01100	Metabolic pathways
ko00940	Phenylpropanoid biosynthesis
ko01110	Biosynthesis of secondary metabolites
ko00941	Flavonoid biosynthesis
ko00480	Glutathione metabolism
ko00950	Isoquinoline alkaloid biosynthesis
ko00053	Ascorbate and aldarate metabolism
ko00350	Tyrosine metabolism
ko04145	Phagosome
ko00908	Zeatin biosynthesis
ko00906	Carotenoid biosynthesis
ko03440	Homologous recombination
ko00072	Synthesis and degradation of ketone bodies

Figure S6 The top 20 KEGG pathways of DEGs in Z-CKL vs Z-TL

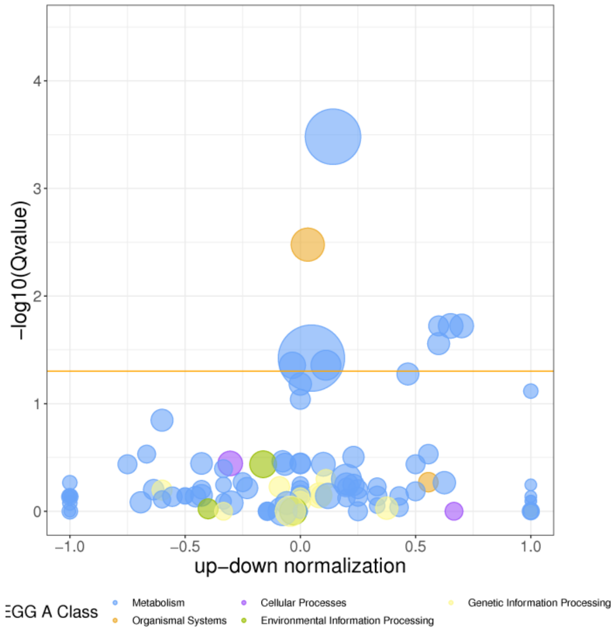

Pathway Top 20

ID	Descrption
ko01110	Biosynthesis of secondary metabolites
ko04626	Plant−pathogen interaction
ko00941	Flavonoid biosynthesis
ko00592	alpha−Linolenic acid metabolism
ko00591	Linoleic acid metabolism
ko00909	Sesquiterpenoid and triterpenoid biosynthesis
ko01100	Metabolic pathways
ko00480	Glutathione metabolism
ko00940	Phenylpropanoid biosynthesis
ko00130	Ubiquinone and other terpenoid−quinone biosynthesis
ko00350	Tyrosine metabolism
ko00254	Aflatoxin biosynthesis
ko00950	Isoquinoline alkaloid biosynthesis
ko00380	Tryptophan metabolism
ko00945	Stilbenoid, diarylheptanoid and gingerol biosynthesis
ko00908	Zeatin biosynthesis
ko00400	Phenylalanine, tyrosine and tryptophan biosynthesis
ko00053	Ascorbate and aldarate metabolism
ko00360	Phenylalanine metabolism
ko04146	Peroxisome

Figure S7 The top 20 KEGG pathways of DEGs in M-TL vs Z-TL

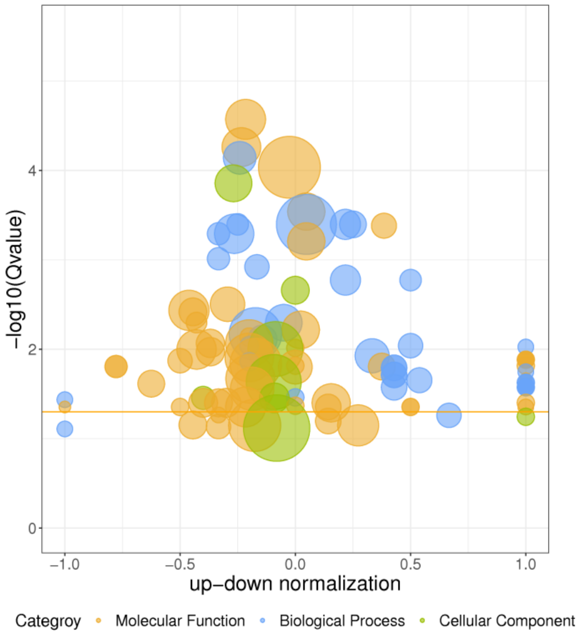

GO term Top 20

ID	Descrption
GO:0016798	hydrolase activity, acting on glycosyl bonds
GO:0004553	hydrolase activity, hydrolyzing O−glycosyl compounds
GO:0000272	polysaccharide catabolic process
GO:0016491	oxidoreductase activity
GO:0005576	extracellular region
GO:0020037	heme binding
GO:0042545	cell wall modification
GO:0045490	pectin catabolic process
GO:0071555	cell wall organization
GO:0055114	oxidation−reduction process
GO:0030599	pectinesterase activity
GO:0045488	pectin metabolic process
GO:0005976	polysaccharide metabolic process
GO:0046906	tetrapyrrole binding
GO:0010393	galacturonan metabolic process
GO:0042737	drug catabolic process
GO:0045229	external encapsulating structure organization
GO:0034754	cellular hormone metabolic process
GO:0005618	cell wall
GO:0015291	secondary active transmembrane transporter activity

Figure S8 The top 20 GO terms of DEGs in M-CKR vs M-TR

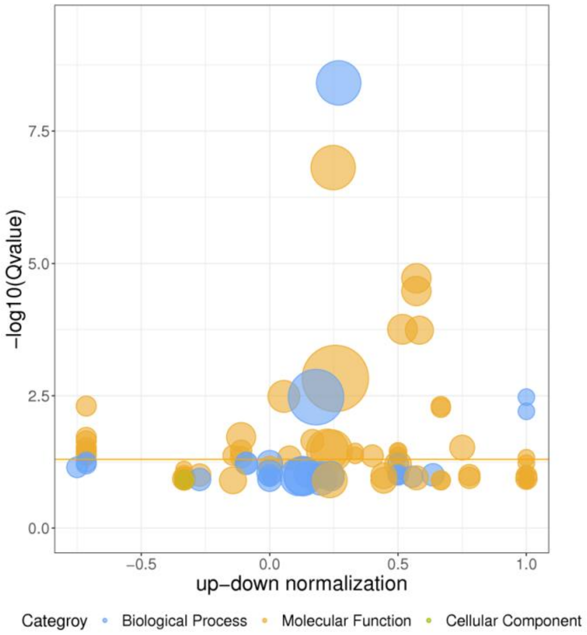

ID	Descrption
GO:0055114	oxidation-reduction process
GO:0016491	oxidoreductase activity
GO:0020037	heme binding
GO:0046906	tetrapyrrole binding
GO:0016705	oxidoreductase activity, acting on paired donors, with incorporation or reduction of molecular oxygen
GO:0005506	iron ion binding
GO:0003824	catalytic activity
GO:0048037	cofactor binding
GO:0044710	single-organism metabolic process
GO:0030212	hyaluronan metabolic process
GO:0048472	threonine-phosphate decarboxylase activity
GO:0018467	formaldehyde dehydrogenase activity
GO:0004400	histidinol-phosphate transaminase activity
GO:1903510	mucopolysaccharide metabolic process
GO:0050662	coenzyme binding
GO:0051903	S-(hydroxymethyl)glutathione dehydrogenase activity
GO:0050607	mycothiol-dependent formaldehyde dehydrogenase activity
GO:0016701	oxidoreductase activity, acting on single donors with incorporation of molecular oxygen
GO:0004029	aldehyde dehydrogenase (NAD) activity
GO:0008194	UDP-glycosyltransferase activity

Figure S9 The top 20 GO terms of DEGs in Z-CKR vs Z-TR

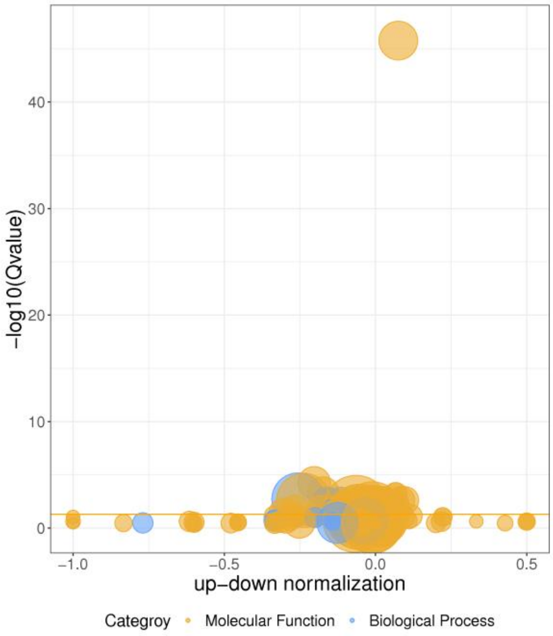

ID	Descrption
GO:0043531	ADP binding
GO:0046906	tetrapyrrole binding
GO:0020037	heme binding
GO:0001871	pattern binding
GO:0030247	polysaccharide binding
GO:0055114	oxidation-reduction process
GO:0048544	recognition of pollen
GO:0009875	pollen-pistil interaction
GO:0009856	pollination
GO:0044706	multi-multicellular organism process
GO:0008037	cell recognition
GO:0016491	oxidoreductase activity
GO:0022838	substrate-specific channel activity
GO:0005216	ion channel activity
GO:0015333	peptide:proton symporter activity
GO:0022897	proton-dependent peptide secondary active transmembrane transporter activity
GO:0015267	channel activity
GO:0022803	passive transmembrane transporter activity
GO:0008194	UDP-glycosyltransferase activity
GO:0016655	oxidoreductase activity, acting on NAD(P)H, quinone or similar compound as acceptor

Figure S10 The top 20 GO terms of DEGs in M-TR vs Z-TR

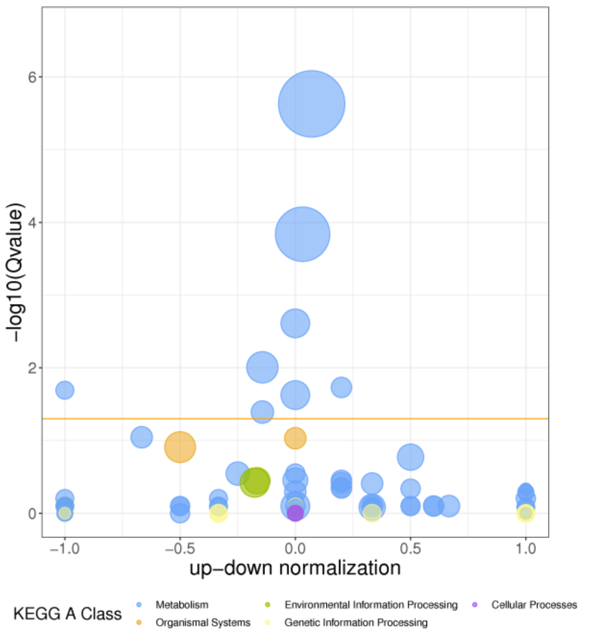

Pathway Top 20

ID	Descrption
ko01100	Metabolic pathways
ko01110	Biosynthesis of secondary metabolites
ko00040	Pentose and glucuronate interconversions
ko00940	Phenylpropanoid biosynthesis
ko00908	Zeatin biosynthesis
ko00944	Flavone and flavonol biosynthesis
ko00500	Starch and sucrose metabolism
ko00909	Sesquiterpenoid and triterpenoid biosynthesis
ko00906	Carotenoid biosynthesis
ko04712	Circadian rhythm – plant
ko04626	Plant–pathogen interaction
ko00010	Glycolysis / Gluconeogenesis
ko00905	Brassinosteroid biosynthesis
ko00710	Carbon fixation in photosynthetic organisms
ko00640	Propanoate metabolism
ko00520	Amino sugar and nucleotide sugar metabolism
ko04016	MAPK signaling pathway – plant
ko04075	Plant hormone signal transduction
ko00100	Steroid biosynthesis
ko00330	Arginine and proline metabolism

Figure S11 The top 20 KEGG pathways of DEGs in M-CKR vs M-TR

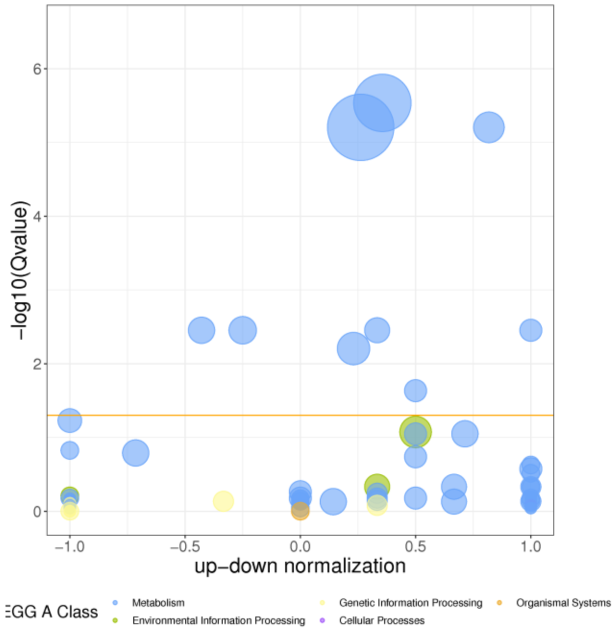

Pathway Top 20

ID	Descrption
ko01110	Biosynthesis of secondary metabolites
ko01100	Metabolic pathways
ko00460	Cyanoamino acid metabolism
ko00350	Tyrosine metabolism
ko00910	Nitrogen metabolism
ko00966	Glucosinolate biosynthesis
ko00592	alpha–Linolenic acid metabolism
ko00940	Phenylpropanoid biosynthesis
ko00904	Diterpenoid biosynthesis
ko00071	Fatty acid degradation
ko04075	Plant hormone signal transduction
ko00270	Cysteine and methionine metabolism
ko00909	Sesquiterpenoid and triterpenoid biosynthesis
ko00531	Glycosaminoglycan degradation
ko00010	Glycolysis / Gluconeogenesis
ko00052	Galactose metabolism
ko00908	Zeatin biosynthesis
ko01210	2–Oxocarboxylic acid metabolism
ko00591	Linoleic acid metabolism
ko00500	Starch and sucrose metabolism

Figure S12 The top 20 KEGG pathways of DEGs in Z-CKR vs Z-TR

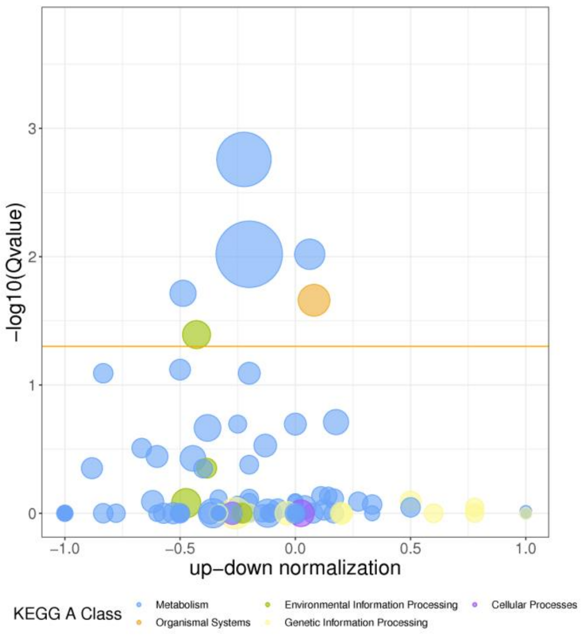

Pathway Top 20

ID	Descrption
ko01110	Biosynthesis of secondary metabolites
ko00940	Phenylpropanoid biosynthesis
ko01100	Metabolic pathways
ko00270	Cysteine and methionine metabolism
ko04626	Plant−pathogen interaction
ko04016	MAPK signaling pathway − plant
ko00906	Carotenoid biosynthesis
ko00904	Diterpenoid biosynthesis
ko00350	Tyrosine metabolism
ko00480	Glutathione metabolism
ko00908	Zeatin biosynthesis
ko00250	Alanine, aspartate and glutamate metabolism
ko00500	Starch and sucrose metabolism
ko00592	alpha−Linolenic acid metabolism
ko00920	Sulfur metabolism
ko00330	Arginine and proline metabolism
ko00010	Glycolysis / Gluconeogenesis
ko00905	Brassinosteroid biosynthesis
ko00052	Galactose metabolism
ko02010	ABC transporters

Figure S13 The top 20 KEGG pathways of DEGs in M-TR vs Z-TR

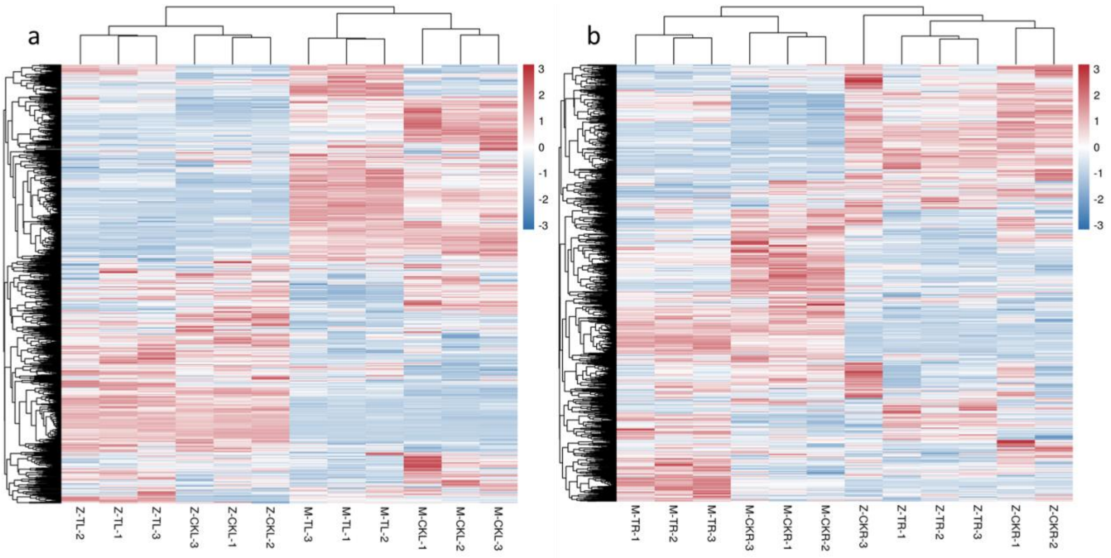

Figure S14 Heatmap of metabolites of leaves and roots in ZM-4 and M9T337 under salt stress. (a) Heatmap of metabolites of leaves in ZM-4 and M9T337 under salt stress. (b) Heatmap of metabolites of roots in ZM-4 and M9T337 under salt stress.

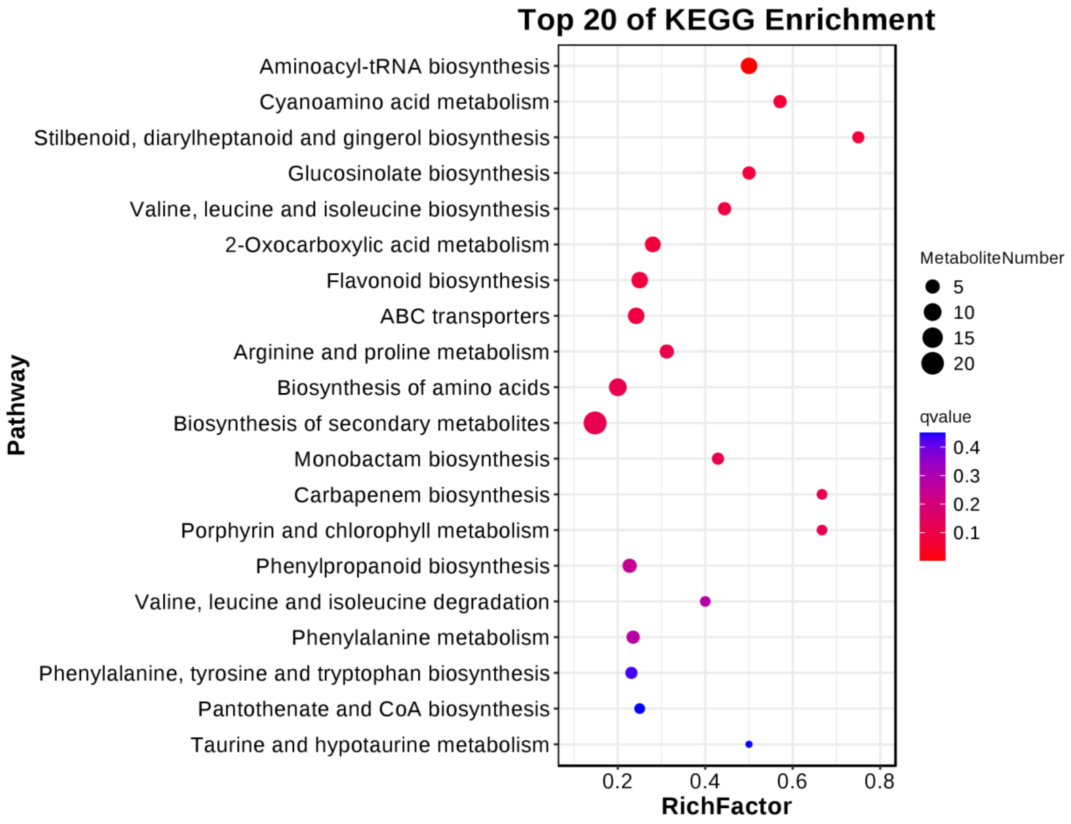

Figure S15 The top 20 KEGG pathways of DAMs in M-CKL vs M-TL

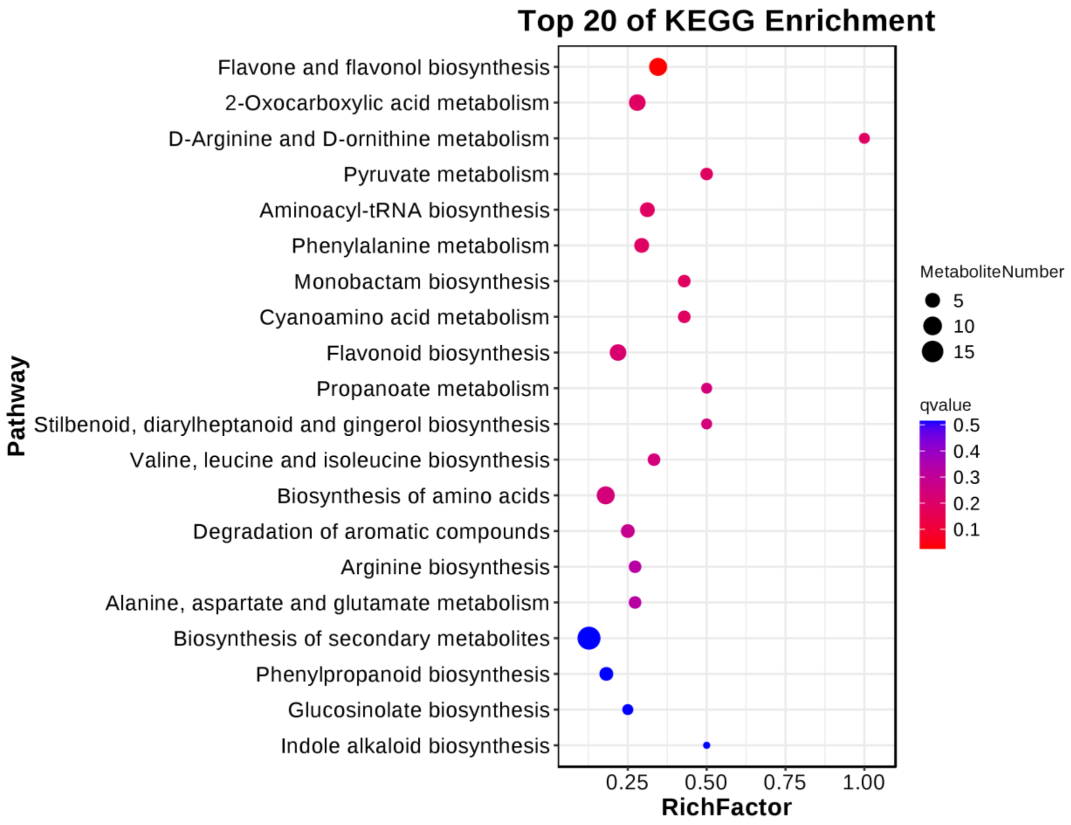

Figure S16 The top 20 KEGG pathways of DAMs in Z-CKL vs Z-TL

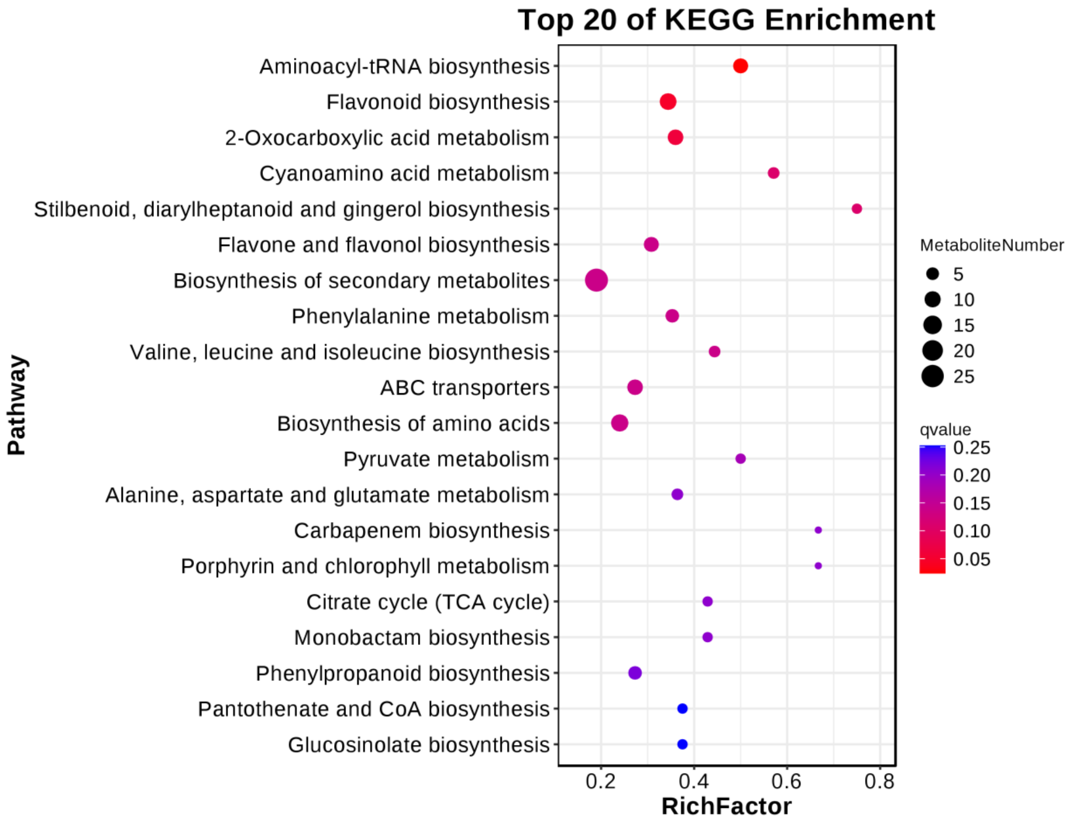

Figure S17 The top 20 KEGG pathways of DAMs in M-TL vs Z-TL

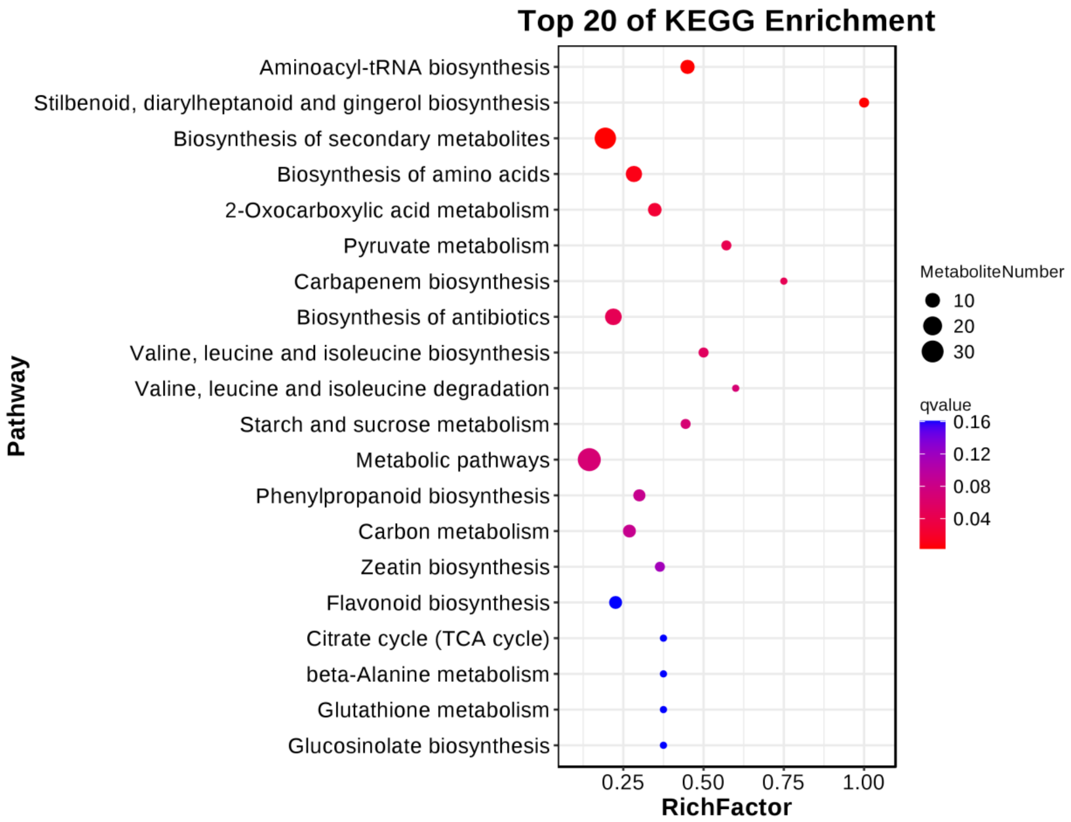

Figure S18 The top 20 KEGG pathways of DAMs in M-CKR vs M-TR

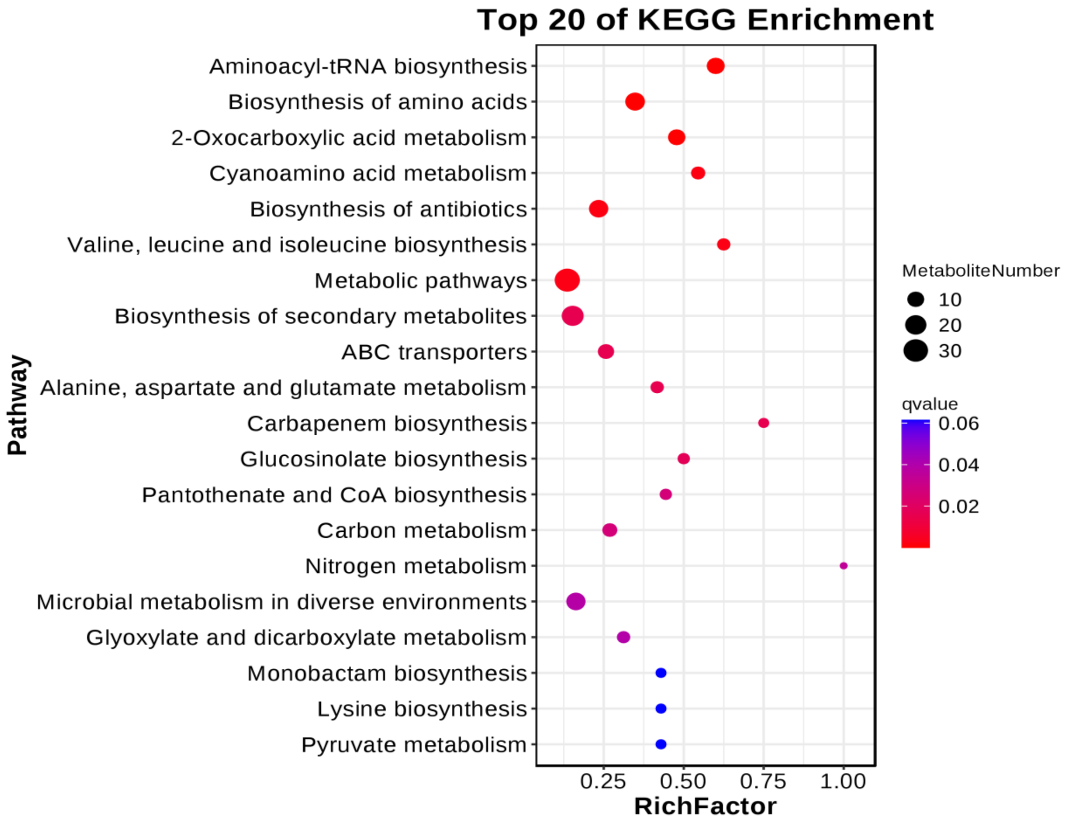

Figure S19 The top 20 KEGG pathways of DAMs in Z-CKR vs Z-TR

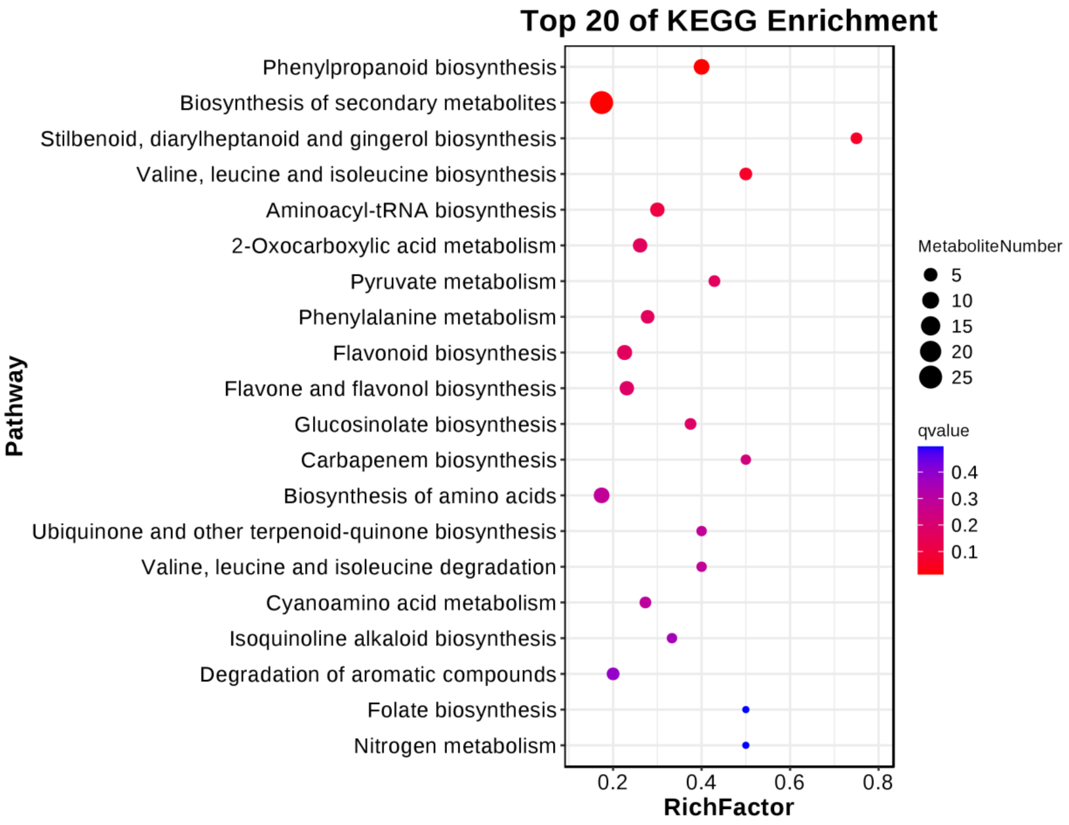

Figure S20 The top 20 KEGG pathways of DAMs in M-TR vs Z-TR

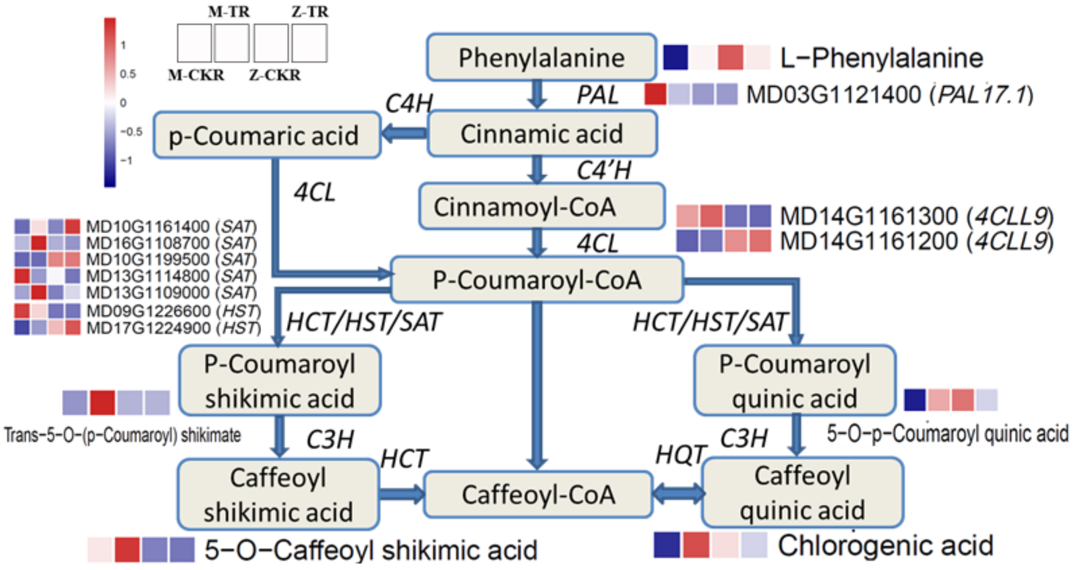

Figure S21 The main salt resistance genes and metabolites in phenylpropanoid biosynthesis pathway (PAL, phenylalanine ammonia-lyase; C4'H, cinnamate-4'-hydroxylase; C4H, cinnamate-4-hydroxylase; 4CL, 4-Coumarate:coenzyme A ligase; HCT/HAT/SAT, Shikimate/Quinate Hydroxycinnamoyltransferase; C3H,p-coumaroyl shikimate 3-hydroxylase; HQT, hydroxycinnamoyl CoA quinate hydrocycinnamoyl transferase).
